# Scalable Fabrication of Large-Scale Electrochromic Smart Windows for Superior Solar Radiation Regulation and Energy Savings

**DOI:** 10.1007/s40820-025-02055-x

**Published:** 2026-01-16

**Authors:** Yanbang Tang, Junyu Yuan, Rongzong Zheng, Chunyang Jia

**Affiliations:** 1 College of Materials and Metallurgy, Guizhou University, Guiyang, 550025 People’s Republic of China; 2 State Key Laboratory of Electronic Thin Films and Integrated Devices, School of Integrated Circuit Science and Engineering, National Engineering Research Center of Electromagnetic Radiation Control Materials, University of Electronic Science and Technology of China, Chengdu, 610054 People’s Republic of China; 3 Shenzhen Institute for Advanced Study, University of Electronic Science and Technology of China, Shenzhen, 518110 People’s Republic of China

**Keywords:** Electrochromic, Smart window, Tungsten oxide, Silver nanowire, Large area

## Abstract

Electrochromic smart windows (ESWs) can significantly reduce building energy consumption, but the high cost hinders large-scale production. The in *situ* growth of tungsten oxide (WO_3_) films is only by a simple immersion process, the silver nanowires (AgNWs) undergo oxidation to Ag^+^ ions through electron loss, and the liberated electrons provide driving force for the deposition of WO_4_^2−^. Enabled the fabrication of large-area WO_3_ films and ESWs were fabricated under minimal laboratory conditions, demonstrating the economic feasibility, efficient and reliable nature of industrial production. Structural characterization and density functional theory calculations were combined to confirm that AgNWs effectively regulate oxygen vacancies of WO_3_ films and promote the in situ growth process. The optimized WO_3_ exhibits a maximum transmittance modulation of 90.8% and excellent cycling stability of 20,000 cycles. The large-scale WO_3_-based ESWs can save building energy up to 140.0 MJ m^−2^ compared to traditional windows in tropical regions, as verified by simulations more than 40 global cities. This research provides a new approach for improving the performance and industrial production of ESW, providing the full understanding and development direction to short the distance of the ESW commercial production.

**Supplementary Information:**

The online version contains supplementary material available at 10.1007/s40820-025-02055-x.

## Highlights

- Propose the in-situ growth strategy of WO_3_ films and deeply explore the growth mechanism and reveal the "one stone, three birds" synergistic mechanism of silver nanowires.
- The WO_3_-based electrochromic devices not only can achieve large-area fabrication, dual-band regulation, and excellent cycling stability, but also possess excellent photothermal control capabilities.
- A uniform and large-scale WO_3_-based electrochromic smart windows of 6000 cm^2^ was fabricated through a simple device, which can save building energy up to 140.0 MJ m^-2^ compared to traditional windows.

**Supplementary Information:**

The online version contains supplementary material available at 10.1007/s40820-025-02055-x.

## Graphical abstract

**Figure:**
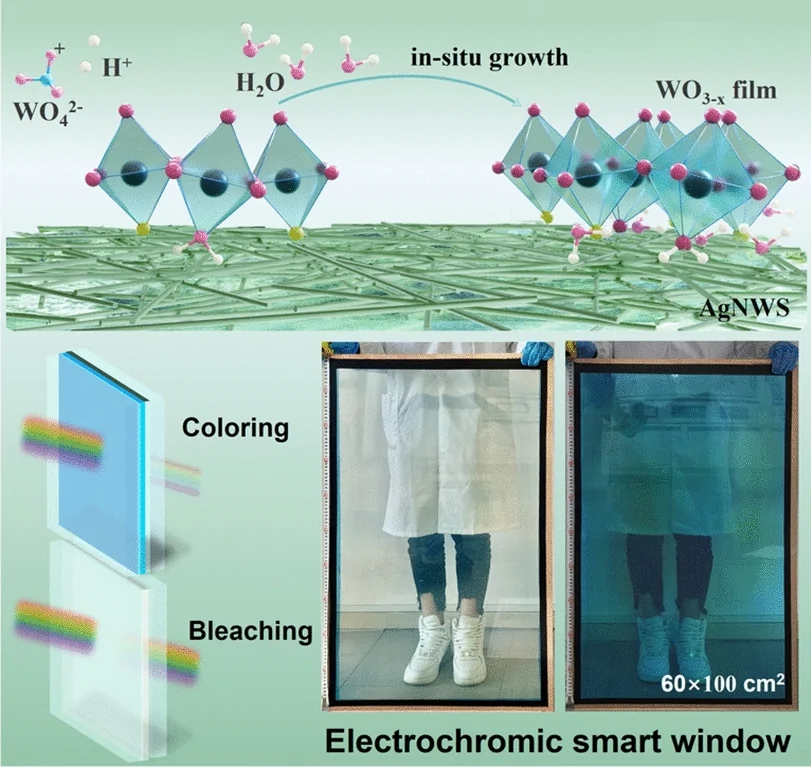

## Introduction

Electrochromism is defined as a reversible modulation of optical properties (e.g., transmittance, absorbance) in electrochromic materials under applied electrical potentials [1, 2]. The emergence of smart building paradigms has driven growing interest in electrochromic materials for advanced fenestration systems [3, 4]. The electrochromic smart window (ESWs) can save about 20%–40% energy consumption in lighting and temperature control of buildings, demonstrating enormous application potential [5–7]. Such smart window technologies enable dynamic control over both visible (VIS) and near-infrared (NIR) spectral regions, thereby synergistically optimizing building energy efficiency through adaptive solar radiation management while maintaining visual comfort [8, 9]. This dual-band engineering approach establishes a fundamental framework for developing next-generation electrochromic smart windows (ESWs) with enhanced spectral selectivity and energy regulation capabilities [10].

Among emerging electrochromic materials, tungsten oxide (WO_3_) has emerged as a prime candidate for ESWs due to its rapid switching kinetics, exceptional optical modulation, and robust cycling stability [11–15]. Nevertheless, the commercial deployment of WO_3_-based ESWs remains impeded by critical bottlenecks, including large-scale limitations in film fabrication, inferior dual-band modulation range, and irreversible regulation capacity decay during prolonged cycling. Conventional WO_3_ film preparation methods, such as hydrothermal synthesis (volume constrained reactors), magnetron sputtering (vacuum chamber size limitations) [16, 17], and electrodeposition (by electrode resistance and precursor solution restricted issues) [18, 19], which invariably suffer from compromised spectral modulation and accelerated performance degradation. Furthermore, the energy-intensive processing of high temperature or pressure conditions associated with these methods not only generates substantial wastewater by-products but also exhibits specific energy consumption, thereby contradicting with the sustainable development of clean energy and responsible production principles.

Recent studies have demonstrated that silver nanowire (AgNWs) electrodes exhibit high transparency, excellent conductivity, and superior mechanical flexibility, along with stable high transmittance in the NIR spectral region [20–26]. The localized surface plasmon resonance (LSPR) effects of AgNWs, observable in both VIS and NIR regions, further enhance their optical modulation. Fan et al. successfully tuned LSPR wavelengths through precise control of AgNWs dimensions and morphology [27]. Zhang et al. fabricated amorphous and porous WO_3_ films capable of dynamic NIR light modulation (780–1850 nm) while maintaining high VIS transparency, achieving through SPR effects [28]. Recent advancements in AgNWs-WO_3_ composite systems have shown that AgNWs integration can enhance the performance of WO_3_-based devices. Such that Hao et al. proposed a WO_3_@AgNW core–shell structure and embedded it in a PDMS substrate to form a “sandwich” embedded electrode, and achieved integration of the conductive layer and the electrochromic layer [29]. Jeon et al. used WO_3_ nanoparticles combined with AgNWs in a simple mixture or in a double-layer structure to enhance the electron/ion transport at the interface between the electrochromic layer and the electrode/electrolyte [30]. However, its optical modulation range and cycle stability are needing improvement.

In this work, WO_3-x_·2H_2_O/AgNWs composite films were successfully prepared via an economical, environmentally friendly, and simple dipping method. Specifically, AgNWs were utilized as sacrificial reducing agents to introduce oxygen vacancies and promote the growth of the films. The optimized films demonstrate exceptional NIR modulation performance and coupled with remarkable cycling stability. Furthermore, we demonstrate the scalable fabrication of ultra-large WO_3-x_·2H_2_O film (100 × 60 cm^2^) and their successful integration into meter-scale ESWs prototypes. Moreover, compared to traditional windows, the ESW can save building energy up to 140.0 MJ m^−2^ by building energy simulations and demonstrated excellent thermal management performance in outdoor testing. This breakthrough establishes a viable pathway for advancing the commercial viability of WO_3_-based ESW technologies.

## Experimental Section

### Materials

Materials: Sodium tungstate dihydrate (Na_2_WO_4_·2H_2_O), citric acid monohydrate, silver nanowires (AgNWs), nitric acid (HNO_3_), ethylene glycol (EG), sodium chloride (NaCl), polyvinylpyrrolidone (PVP), silver nitrate (AgNO_3_), propylene carbonate (PC), lithium perchlorate (LiClO_4_), acetone, and ethanol are all purchased at Aladdin or the Discovery Platform for direct use without purification. Fluorine-doped tin oxide glass (FTO) with a thin layer resistance of 15 Ω cm^−2^ was purchased from the market and sonicated in detergent, isopropanol, distilled water, and ethanol for 30 min. After drying in a 50 °C oven, a clean fluorine-doped tin oxide (FTO) substrate is prepared after an additional 40 min of ozone cleaning.

### Preparation of Silver Nanowires

The 1.86 g of PVP is added to 100 mL of EG and stir until evenly dissolved at 160 °C and then drop in a 1 mL of 3 M NaCl, named A solution. Completely coat the beaker with tin foil, and the 0.5 g of AgNO_3_ is added to 25 mL of EG and sonicated for 1 h, named B solution. Finally, the B solution was slowly added to the A solution and stirred for 1.5 h, and the solution changed from colorless and transparent to gray-green oily; wait for the solution to cool to room temperature, wash with acetone and ethanol for several centrifugations, and finally obtain ethanol-dispersed AgNWs (Fig. S1).

### Preparation of WO_3_ Precursor Solution

Weigh 0.3 g of Na_2_WO_4_·2H_2_O and dissolve it in 30 mL of deionized water. Subsequently, 0.6 g of citric acid is added to the solution and ensure complete dissolution. Finally, 3.5 mL of 3 M HNO_3_ was added. The resulting mixture is stirred for 20 min to obtain a WO_3_ precursor solution.

### Preparation of WO_3-x_·2H_2_O/AgNWs Films

First, one end of the FTO substrate is carefully covered with 1-cm-wide polyimide tape, leaving a portion of the conductive surface for subsequent performance testing. AgNWs were prepared into 0.5, 1.0, and 1.5 mg mL^−1^, and then, different concentrations of AgNWs were spun coated onto the FTO substrate and dried in an oven for 5 min. Then, pour a certain amount of WO_3_ precursor solution into a beaker and immerse the dry AgNWs/FTO substrate in the precursor solution so that the conductive side is facing down. Heat in a 60 °C water bath for 30 min, remove FTO from the beaker for rinsing, and remove the sediment on the back. After drying, the A_x_-W films are obtained, where x represents the concentrations of AgNWs at 0.0, 0.5, 1.0, and 1.5 mg mL^−1^.

### Fabrication of the Electrochromic Devices (ECDs)/ESWs

Fabrication of the ECDs/ESWs: Small electrochromic devices (ECDs) with assembled sizes of 2 × 4 cm^2^ are used for performance testing, while large electrochromic smart windows (ESWs) with construction sizes of 10 × 10 and 100 × 60 cm^2^ are used for commercial feasibility verification. Large-size A_x_-W films and large-size Prussian blue films were prepared by using a self-made large-scale water bath heating device, and the operation can be found in the Supporting Information. Subsequently, the two are assembled to form an electrochromic smart window. These devices use A_1.0_-W films as the cathode and Prussian blue film as the anode, and 2 × 4 cm^2^ and 10 × 10 cm^2^ devices use 1 M LiClO_4_/PC as the electrolyte, and 100 × 60 cm^2^ large-size devices use an all-solid-state electrolyte.

## Results and Discussions

### Growth Mechanism and Characterization of WO_3-x_·2H_2_O Film

**Fig. 1:**
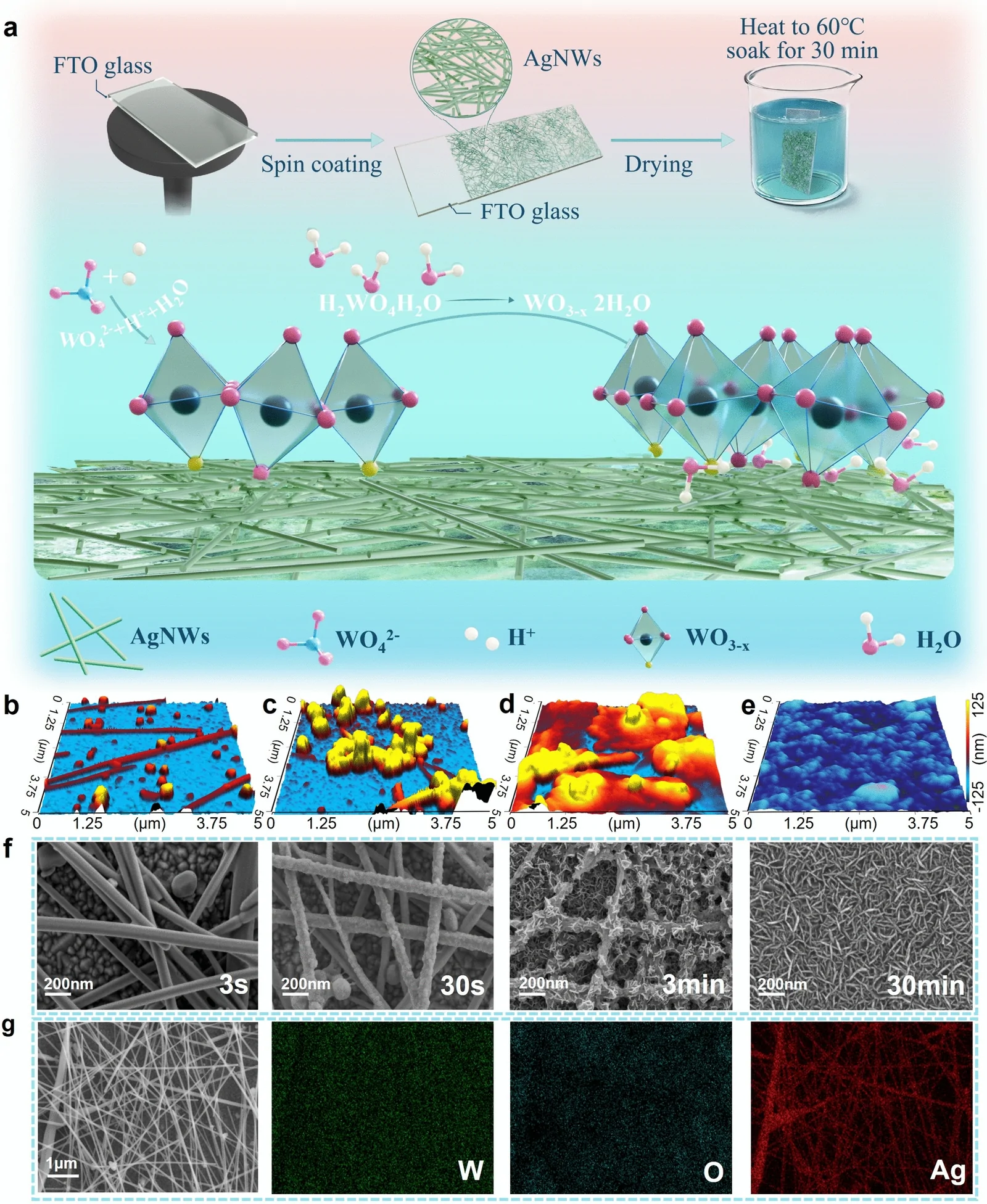
**a** Schematic diagram of the preparation process and growth mechanism of WO_3-x_·2H_2_O film. **b**–**e** Nano-infrared spectra of A_1.0_-W film at different growth times. **f** SEM image of A_1.0_-W film at different growth times. **g** EDS image of A_1.0_-W film with 3 s

The process and mechanism of the preparation of WO_3-x_·2H_2_O film is illustrated in Fig. 1a. The average length of the self-prepared AgNWs is approximately 5 μm, with a diameter of about 40 nm (Fig. S1). Uniform WO_3-x_·2H_2_O films were prepared via a simple spin-coating and simple immersion process. With AgNWs concentrations of 0.0, 0.5, 1.0, and 1.5 mg mL^−1^, the prepared WO_3-x_·2H_2_O/AgNWs films were labeled as A_0.0_-W, A_0.5_-W, A_1.0_-W, and A_1.5_-W, respectively as shown in Fig. S2. The A_0.0_-W film appears pale yellow, whereas the A_0.5_-W, A_1.0_-W, and A_1.5_-W films display a light blue hue. This color shift likely arises from AgNWs accelerating the growth rate during film formation, which promotes electron accumulation and induces oxygen vacancy formation in the WO_3_ crystal structure [11].

Initial scanning electron microscopy (SEM) characterization was performed to evaluate the impact of AgNWs incorporation for the WO_3_ film formation kinetics preliminarily (Fig. S3). The A_0.0_-W film exhibited delayed nucleation, with no significant morphological changes observed within the first 5 min of growth, followed by the emergence of sparse WO_3_ structures after 10 min. In contrast, the A_1.0_-W film forming well-defined WO_3_ nanosheets within just 5 min of immersion growth. These results indicate that AgNWs significantly accelerate the nucleation and growth rate of WO_3_ film. A short growth time (10 or 20 min) resulted in incomplete WO_3_ film formation with discontinuous surface coverage, while excessively long growth times (40 or 50 min) produced overly thick film, inducing stress at the film-substrate interface (Fig. S3). Consequently, the growth time of 30 min was found to produce continuous and homogeneous WO_3_ film. Furthermore, cross-sectional images also confirmed this conclusion (Fig. S4).

The early-stage growth process of the A_1.0_-W film was systematically analyzed through nano-infrared spectroscopy and SEM characterization. The elemental distribution where the orange-red signals correspond to silver (Ag) and deep-blue regions represent tungsten (W) as shown in Fig. 1b–e, captured at progressive growth durations of 3 s, 30 s, 3 min, and 30 min, respectively. The nano-infrared spectra reveal the detailed growth process, and Ag elements gradually decompose and diffuse from the aggregated AgNWs, as illustrated in Fig. 1c, d, concurrent with the rapid nucleation and growth of the WO_3_. Ultimately, the WO_3_ grows to a certain thickness and completely covers the initial AgNWs substrate after 30 min. The morphological film of the different growth times was also captured by SEM (Fig. 1f). The further EDS analysis of films with varying growth duration demonstrates that prolonged growth duration induces progressive Ag depletion with enhanced dispersion, while W concentration increases concomitantly through preferential accumulation around AgNWs templates (Figs. 1g and S5). The morphological evolvement and elemental redistribution were accorded with the result of the nano-infrared spectra, which the WO_3_ nucleation and growth process coupled with the AgNWs decompose and Ag element diffuse. So, the hypothesis of the WO_3_ film growth mechanism could proceed through two distinct yet synergistic pathways.

Firstly, AgNWs function as sacrificial reducing agents in the reaction process. In the nitric acid (HNO_3_) medium, Ag undergoes oxidation to Ag^+^ ions through electron loss. The liberated electrons (e^−^) are efficiently transported via the conductive AgNWs/FTO network to the substrate interface. This electron transfer creates a localized high electron density region at the interface, which provides a driving force for the second growth stage of the WO_3_ film, as shown in Eq. (1). To verify the consumption of AgNWs, Ag element absorption test was conducted on the precursor solution after 5 min of reaction via flame atomic absorption spectrometry. The result showed that the concentration of Ag element in the solution was 0.084 mg L^−1^, and this result provides direct evidence for the consumption of AgNWs.

1 $${\text{Ag }} + {\text{ HNO}}_{{3}} \to {\text{ AgNO}}_{{3}} + {\text{ H}}^{ + } + {\text{ e}}^{ - }$$

**Fig. 2:**
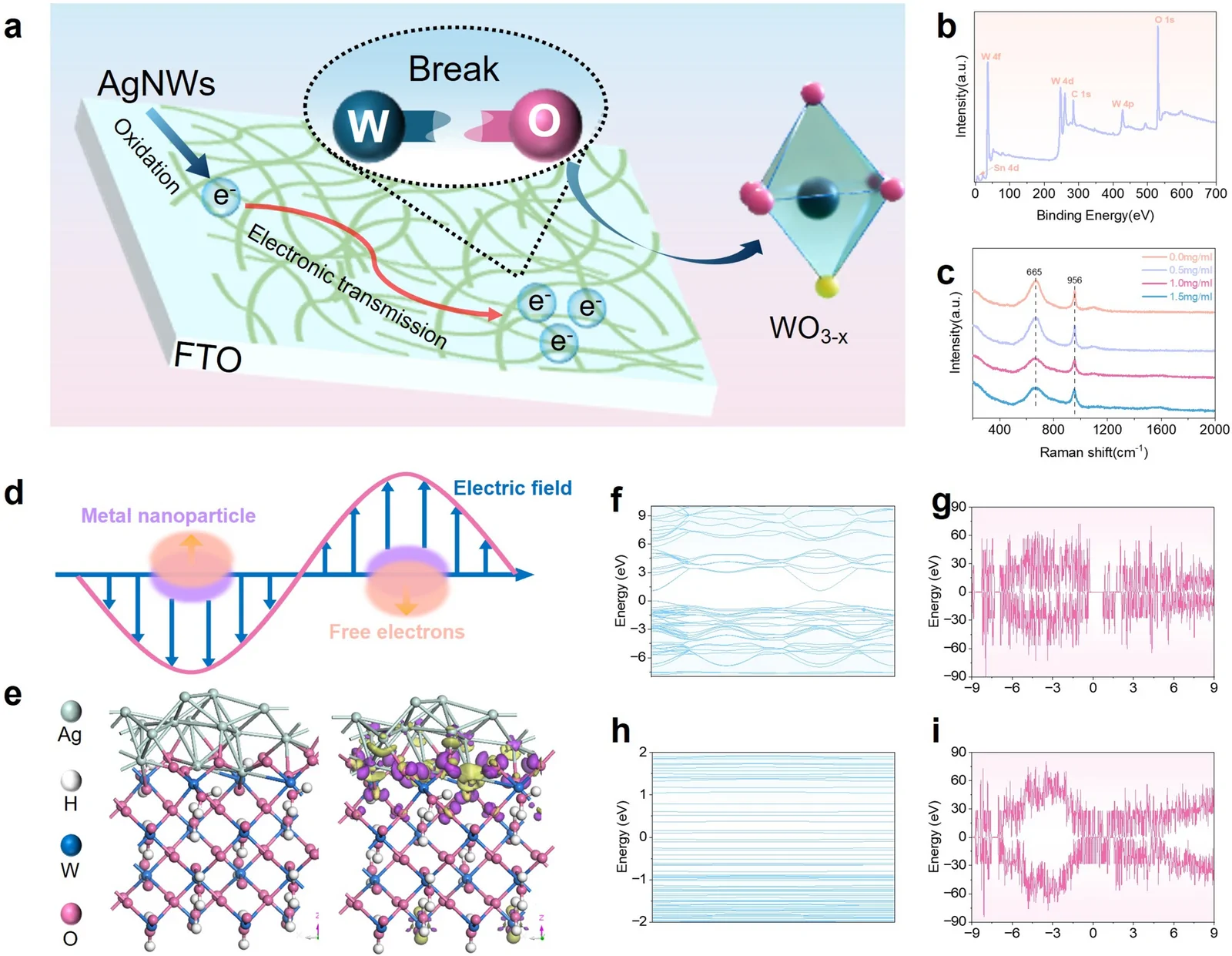
**a** WO_3_ film growth mechanism. **b** XPS full spectrum of A_1.0_-W film. **c** Laser Raman pattern of A_x_-W films. **d** LSPR schematic. **e** Crystal structure and deformation charge density map of AgNWs and WO_3_·2H_2_O. **f**–**g** Band structure and density of states of A_0.0_-W film. **h**–**i** Band structure and density of states of A_1.0_-W film

Secondly, the e^−^ generated in the first stage participates in the deposition of WO_4_^2−^ species in the precursor solution under acidic conditions, breaking the W=O bond and forming oxygen vacancies (Fig. 2a). Among them, the oxygen vacancy is positive, maintaining electrical neutrality by adsorbing OH^−^ or H_2_O in the solution, while enhancing the hydrophilicity and ion exchange capacity of the material. Ultimately, it is formed of non-stoichiometric WO_3-x_·nH_2_O film, as shown in Eq. (2):

2 $${\mathrm{WO}}_{{4}}^{{{2} - }} + {\text{ nH}}^{ + } + {\text{ ne}}^{ - } \to {\text{ WO}}_{{{3} - {\mathrm{x}}}} \cdot{\mathrm{nH}}_{{2}} {\text{O }} + \, \left( {{1} - {\mathrm{x}}} \right){\text{ H}}_{{2}} {\mathrm{O}}$$

To further confirm inferred growth mechanism of WO_3-x_·nH_2_O film, the detailed structural characterizations of the A_1.0_-W film were conducted. The X-ray diffraction (XRD) pattern of the A_1.0_-W film is shown in Fig. S6. The diffraction peaks show excellent agreement with standard WO_3_·2H_2_O (JCPDS No. 18–1420). Peaks from the FTO substrate (JCPDS No. 41–1445) were also observed. These diffraction peaks confirm the successful preparation of A_x_-W films. Notably, no Ag-related peaks were detected, likely due to the AgNWs concentration being below the XRD detection limit. Further structural analysis was performed using transmission electron microscopy (TEM) on the A_1.0_-W film. The EDS spectrum (Fig. S7a) confirms the presence of residual AgNWs in the composite film, indicating trace Ag incorporation despite its absence in XRD patterns. High-resolution TEM revealed lattice fringes with interplanar spacings of 0.377, 0.370, and 0.184 nm, matching the (200), (001), and (002) planes of WO_3_·2H_2_O, respectively (Fig. S7b). This is further supported by the selected area electron diffraction (SAED) pattern (Fig. S7c), which shows distinct diffraction rings indexed to these crystal planes.

Laser Raman spectroscopy and X-ray photoelectron spectroscopy (XPS) analyses were conducted on the A_x_-W films. The full XPS spectrum of the A_1.0_-W film (Fig. 2b) shows no detectable Ag-related signals, consistent with XRD results, confirming that AgNWs concentrations fall below the detection limits of both techniques. The O 1*s* XPS spectra of all A_x_-W films reveal three characteristic peaks of 530.2 eV (W–O), 531.4 eV (OH), and 529.3 eV (H_2_O) [31], confirming structural water content in alignment with XRD results (Fig. S8). The XPS spectra of W 4*f* present in Fig. S9a-d, where the peaks at 35.4 and 37.6 eV correspond to W 4*f*_7/2_ and W 4*f*_5/2_, is related to W^6+^ in the WO_3_·2H_2_O phase. The peaks at 34.2 and 36.5 eV can be attributed to W^5+^ 4*f*_7/2_ and W^5+^ 4*f*_5/2_ [31–37], indicating the presence of oxygen vacancies in the film. Notably, the intensity of the 36.5 eV peak increases with AgNWs concentration, further supporting the proposed growth mechanism. As displayed in Fig. 2c, two distinct vibrational bands at 665 and 956 cm^−1^ were detected from Laser Raman spectroscopy, which corresponds to the stretching mode of O-W–O and arises from the terminal bond W=O [38, 39], respectively. Both vibrational modes are clearly resolved in the A_x_-W films. Notably, the width of peak at 665 cm^−1^ progressively increases with higher AgNWs concentrations. This peak broadening is consistent with enhanced oxygen vacancy concentrations [40–42], as confirmed by complementary XPS detection of W^5+^ species. The combined Raman and XPS results provide mutually consistent evidence for oxygen vacancy formation in the A_x_-W films.

The crystal structure and elemental composition of the A_x_-W films were characterized using the material analysis techniques outlined above. Subsequent investigations explored the influence of AgNWs on the optical properties of the film. When light interacts with AgNWs, the electromagnetic field perturbs their electron cloud distribution, displacing electrons from equilibrium positions and inducing collective oscillations (Fig. 2d). This interaction generates the LSPR effect through amplified electromagnetic field enhancement at the AgNWs surface, significantly boosting light absorption at resonant wavelengths [43].

To quantify this phenomenon, UV–VIS absorption spectroscopy was performed on films with varying AgNWs concentrations (Fig. S10). The spectra revealed a distinct absorption maximum at 390 nm, with peak intensity demonstrating a linear correlation to AgNWs loading. This direct proportionality confirms that increased AgNWs content amplifies the LSPR effect at this wavelength. The absorption spectrum and corresponding Tauc plot of A_x_-W films are shown in Fig. S11. The A_0.5_-W, A_1.0_-W, and A_1.5_-W films demonstrate dual absorption bands at 313 and 1100 nm. The 313 nm peak corresponds to the intrinsic absorption of WO_3-x_·2H_2_O [44], while the 1100 nm feature is attributed to the LSPR of AgNWs. Notably, the LSPR absorption intensity at 1100 nm in A_0.5_-W, A_1.0_-W, and A_1.5_-W films shows a direct correlation with AgNWs concentration, suggesting enhanced plasmonic coupling effects at higher nanofiller loadings. Additionally, we calculated the optical bandgap of A_x_-W films using Eq. (3) based on the WO_3-x_·2H_2_O absorption edge, as illustrated in Fig. S11.

3 $$\left( {\alpha {\mathrm{hv}}} \right)^{{{1}/{\mathrm{n}}}} = {\text{ A }}\left( {{\text{hv }} - {\text{ Eg}}} \right)$$

where α represents the absorption coefficient, A denotes a proportionality constant, and n indicates the transition type parameter (with n = 1/2 for direct bandgap semiconductors and n = 2 for indirect bandgap semiconductors). Given that WO_3-x_·2H_2_O is an indirect bandgap semiconductor, n is taken to be 2. Through this analysis, the optical bandgaps of A_0.0_-W, A_0.5_-W, A_1.0_-W, and A_1.5_-W films were determined to be 3.02, 2.99, 2.97, and 2.94 eV, respectively. These values establish a systematic reduction in optical bandgap with increasing AgNWs concentration in the A_x_-W films series.

The growth mechanism of A_x_-W films was further elucidated through density functional theory (DFT) calculations. Charge transfer dynamics at the AgNWs/WO_3_·2H_2_O interface were investigated through differential charge density analysis, where the purple (Δρ < 0) and yellow (Δρ > 0) represent electron accumulation and depletion regions, respectively, as shown in Fig. 2e. It was observed that AgNWs transfer electrons to WO_3_·2H_2_O and alter the electronic structure of WO_3_·2H_2_O, which is consistent with Eq. (1) assumed growth mechanism. Electron injection causes the Fermi level of WO_3_·2H_2_O to shift toward the conduction band, resulting in changes in the density of electronic states, which in turn affects the band structure. Additionally, this electron accumulation enhances the reactivity of the WO_3_·2H_2_O surface and reduces the reaction barrier for subsequent deposition, promoting the effective deposition of WO_3_·2H_2_O onto the substrate and accelerating its growth, consistent with the growth process discussed in Fig. S3 earlier.

To verify whether the band structure of WO_3_·2H_2_O changes, band structure and density of states (DOS) calculations were performed on A_0.0_-W film and A_1.0_-W film, and the results are shown in Fig. 2f–i. The band structure of A_0.0_-W and A_1.0_-W film is depicted in Fig. 2f, h, revealing that the band gap of WO_3-x_·2H_2_O decreased from 1.02 to 0.15 eV. It attributed to the transfer of electrons from AgNWs to WO_3_·2H_2_O, which fills the bottom of the conduction band with additional electrons, thereby altering the density of electronic state. The DOS analysis, as shown in Fig. 2g, i, clearly demonstrates that the electron transfer from AgNWs introduces new electronic states within the original bandgap of WO_3_·2H_2_O, while simultaneously raising the Fermi level toward the conduction band. The proposed growth mechanism and hypotheses were further validated through computational DFT analysis, demonstrating excellent agreement between theoretical predictions and experimental observations.

In summary, the WO_3-x_·2H_2_O film was successfully prepared using the simple immersion process, through comprehensive material characterization, and DFT calculations have elucidated the growth mechanism and demonstrated the significant influence of AgNWs on the film.

### Electrochemical Properties of the WO_3-x_·2H_2_O Film

**Fig. 3:**
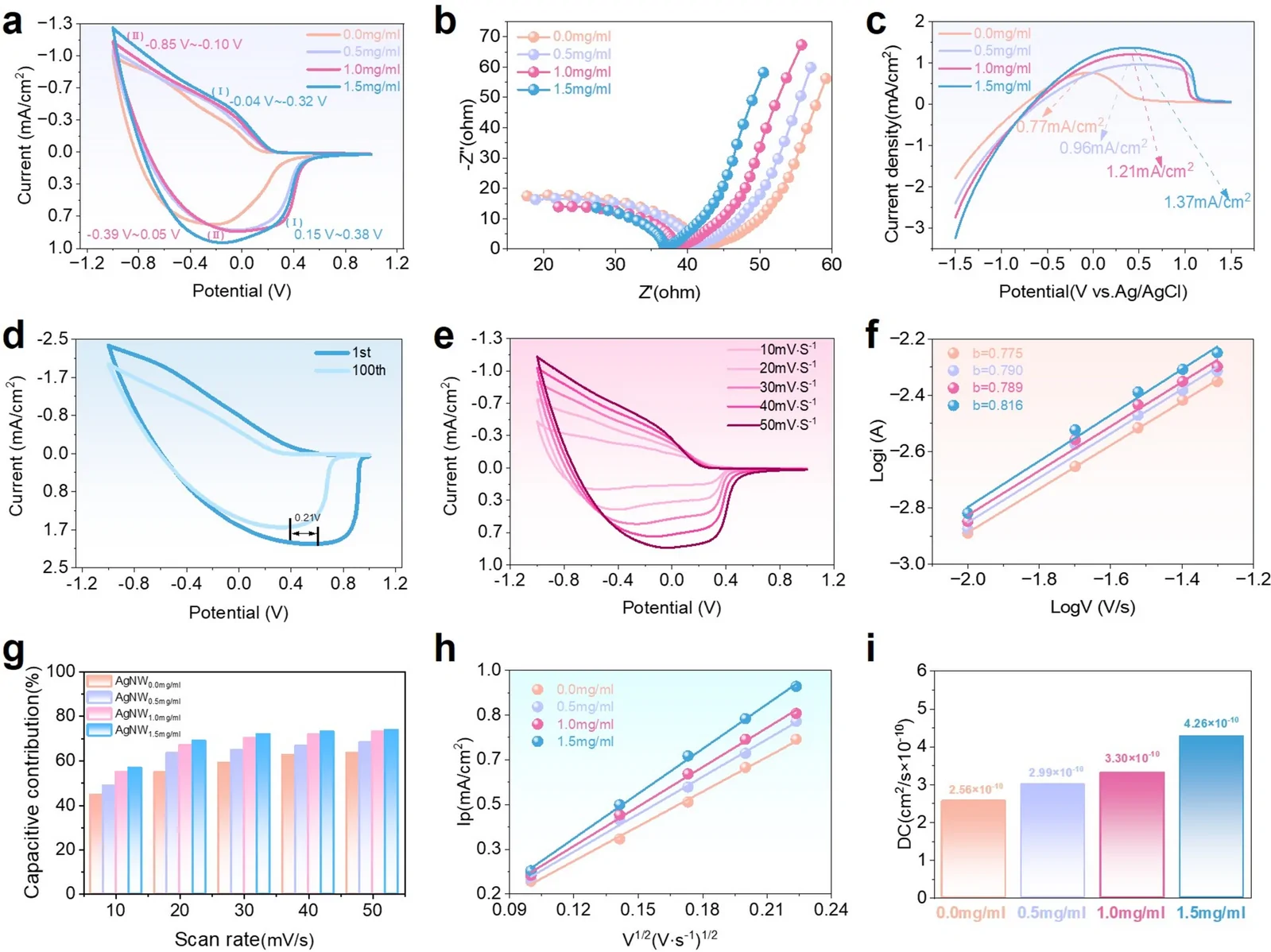
**a** CV plot of A_x_-W films. **b** Nyquist plot of A_x_-W films. **c** Current density–voltage curve of A_x_-W films. **d** 100-turn CV cycle test plot of A_1.0_-W film. **e** CV plot of different scan rates of A_1.0_-W film. **f** Relationship between log v and log i. **g** Percent change in capacitance behavior at different scan rates. **h** Peak current density vs. square root of scan rate. **i** Diffusion coefficient plot of A_x_-W films

The electrochemical performance of the A_x_-W films was evaluated using a three-electrode system. As shown in Fig. 3a, the cyclic voltammetry (CV) curves of A_x_-W films with different AgNWs concentrations exhibit increasing enclosed areas with higher AgNWs content. This phenomenon is primarily attributed to the elevated concentration of AgNWs, which substantially enhances the electronic conductivity of the film. Secondly, the increased concentration of oxygen vacancies not only accelerates the carrier diffusion rate but also provides additional reactive active sites. Finally, as the film thickness increases, the total amount of active substances involved in the electrochemical reaction is correspondingly augmented. Consequently, the expansion of the closed area in CV curves arises from the synergistic effects of three factors: the improved macroscopic conductivity of the electrode, the enhanced intrinsic activity of the material at the microscale, and the increased total amount of active substances. Two pairs of redox peaks are observed in Fig. 3a, labeled as (I) and (II), corresponding to two lithium insertion processes, representing the conversion steps from W^6+^ to W^5+^ and from W^5+^ to W^4+^, respectively [45, 46]. The main electrochromic behavior of the A_x_-W films derives from the transition of W^4+^ at peak (II), while the coloration efficiency related to the transition of W^5+^ at peak (I) is relatively low during the electrochromic process [47]. Although the increase at peak (I) indicates enhanced lithium insertion, the optical changes it induces are weak, reducing the efficiency of the electrochromic process in the A_x_-W films [11].

The electrochemical impedance spectroscopy (EIS) was employed to assess the ionic transport kinetics at the interface between the film electrochromic layer and the electrolyte. Figure 3b presents Nyquist plots for different A_x_-W films, and the equivalent circuit was depicted in Fig. S12. The charge transfer resistance (R_ct_) reflects the charge transfer difficulty at the electrode–electrolyte interface. The high conductivity of AgNWs and the oxygen vacancies in the films enhance the charge transport properties [48, 49], thereby reducing the R_ct_ of the films. The current density–voltage (I-V) curves of A_x_-W films revealed the voltage-dependent current response as shown in Fig. 3c, and the current density increases with higher AgNWs concentration, indicating improved conductivity and charge carrier density [50]. This observation aligns with the results of EIS, both reflecting enhanced conductivities.

The electrochemical stability of the A_x_-W films was evaluated by CV measurements. As illustrated in Figs. 3d and S13, the CV enclosed area retention rates of the A_0.0_-W, A_0.5_-W, A_1.0_-W, and A_1.5_-W films were 62.2%, 66.3%, 67.6%, and 67.4% after 100 cycles, respectively. A comparative analysis demonstrated that the A_1.0_-W film showed the smallest decrease in CV curve integration area. The peak potential difference of the A_1.0_-W film was found to be the smallest, which was calculated by selecting the corresponding potentials at the current extreme points of the CV curves before and after 100 cycles. This phenomenon can be attributed to the A_0.5_-W, A_1.0_-W, and A_1.5_-W films which contain unconsumed AgNWs that serve as a secondary conductive layer to enhance electrical conductivity and form a stable conductive network (Fig. S14). Moreover, the oxygen vacancies in the films can act as reaction active sites, which also makes the insertion/extraction process more reversible and reduces the internal stress caused by repeated volume changes, which helps maintain the integrity of the crystal structure and reduce attenuation. However, the A_1.5_-W film exhibits significant degradation due to excessive thickness (Fig. S15d). The increased thickness leads to prolonged ion diffusion paths and compromised structural stability, further exacerbating its performance decay. Consequently, optimal AgNWs concentration is crucial for fabricating film with superior performance.

To further investigate the charge transport dynamics, CV measurements of the A_x_-W films were performed at various scan rates. As depicted in Figs. 3e and S16, both anodic and cathodic peak currents exhibit an increase with rising scan rates, indicating dominant capacitive behavior in the electrochemical process [51]. The scan rate-dependent peak current variations were quantitatively analyzed using Eq. (4) to elucidate the charge storage mechanism.

4 $${\text{i }} = {\text{ av}}^{{\mathrm{b}}}$$

where a and b are variables. The value of b can be determined by fitting the log i and log v relations into a straight line, where a value of 0.5 represents diffusion-controlled charge transport and a value of 1 represents the dominance of capacitive behavior. As shown in Fig. 3f, the calculated values b for A_0.0_-W, A_0.5_-W, A_1.0_-W, and A_1.5_-W films are 0.76, 0.78, 0.81, and 0.82, respectively. This indicates that the ions in the A_x_-W films are controlled by diffusion-controlled and capacitive-controlled mixing processes. Moreover, with the increase in AgNWs concentration, the charge transport process of the film is more capacitively controlled. Equation (5) is used to quantitatively study the contribution of diffusion control and capacitance control to electrochemical processes at different scan rates.

5 $${\mathrm{i}}\left( {\mathrm{v}} \right) \, = {\text{ k}}_{{1}} {\text{v }} + {\text{ k}}_{{2}} {\mathrm{v}}^{{{1}/{2}}}$$

k_1_v and k_2_v^1/2^ represent the capacitance control part and the diffusion control part, respectively. The scan rate-dependent capacitive contribution percentages are plotted in Fig. 3g, revealing increasingly dominant capacitive behavior at higher scan rates [52], demonstrating the growing influence of capacitive control in the electrochemical process. Notably, higher AgNWs concentrations consistently yield greater capacitive contributions across all scan rates, indicating significantly enhanced capacitive behavior [45]. To gain insight into the diffusion kinetics of ions in A_x_-W films, the diffusion coefficients were calculated using the Randles–Sevcik Eq. (6).

6 $${\mathrm{I}}_{{\mathrm{p}}} = { 269}000{\mathrm{n}}^{{{3}/{2}}} {\mathrm{AC}}_{0} {\mathrm{D}}^{{{1}/{2}}} {\mathrm{v}}^{{{1}/{2}}}$$

where I_p_ represents the peak current, n denotes the number of electrons transferred during redox, A is the electrode area, D signifies the diffusion coefficient, C_0_ indicates the electrolyte concentration, and v corresponds to the scan rate. Figure 3h reveals a distinct linear correlation between peak current density and the square root of scan rate, facilitating precise determination of diffusion coefficients through the Randles–Sevcik equation. As shown in Fig. 3i, with higher AgNWs concentration, the diffusion coefficient increases, which is in agreement with the observed capacitive behavior dominance [53].

### Electrochromic Operation Performance of WO_3-x_·2H_2_O Film

**Fig. 4:**
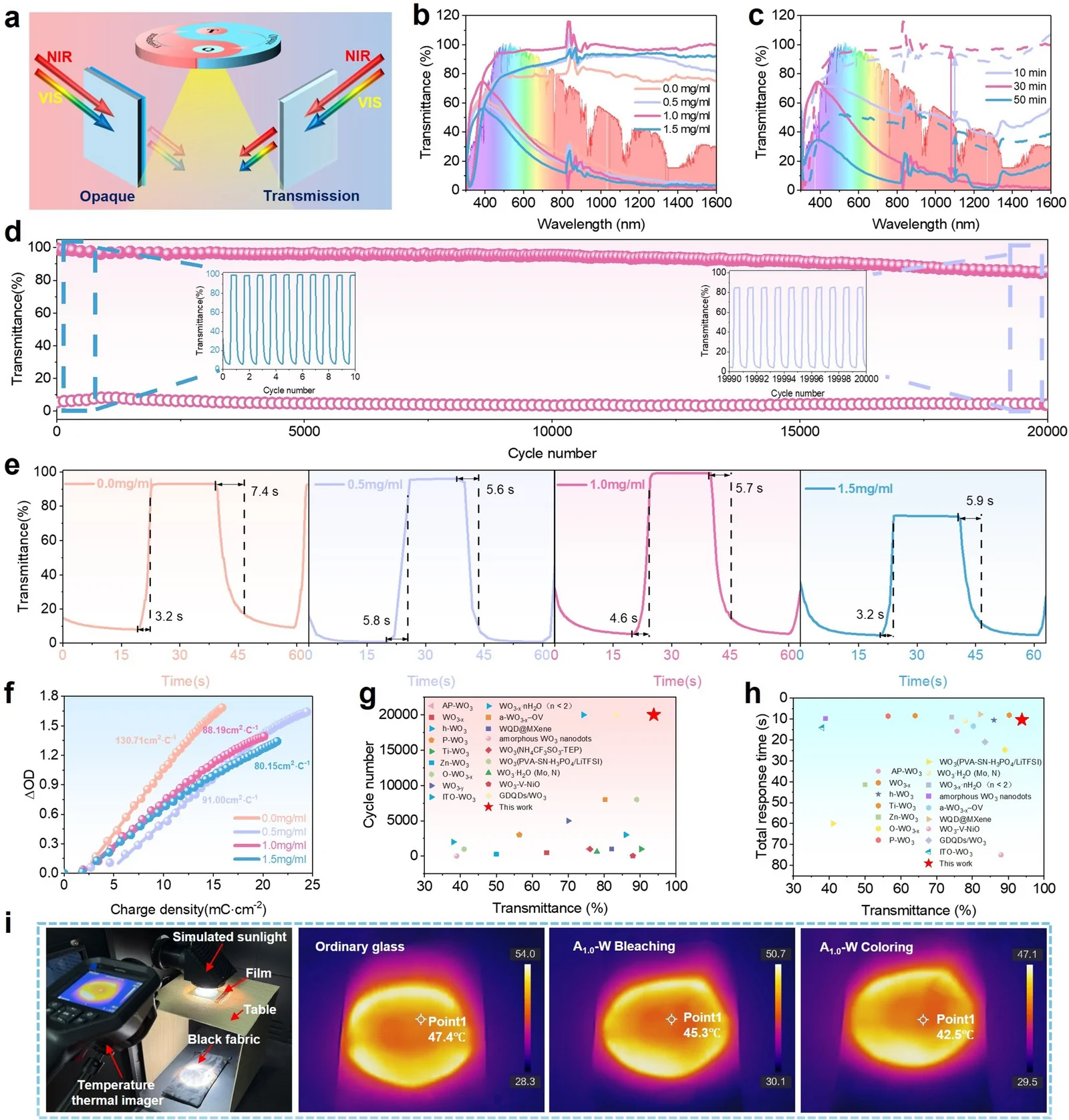
**a** Electrochromic schematic. **b** Transmittance curve of A_x_-W films. **c** Transmittance curve of A_1.0_-W film with different growth times. **d** Transmittance and number of cycles of A_1.0_-W film. **e** Response time plot of A_x_-W films. **f** Coloration efficiency plot of A_x_-W films at 1100 nm. **g**–**h** Performance comparison chart of WO_3_-based electrochromic films in recent years. **i** A_1.0_-W film solar light simulation data graph

The A_x_-W films investigated represents a typical cathodic electrochromic material, with its operational mechanism illustrated in Fig. 4a. The dynamic transmittance spectra in the 300–1600 nm range are presented in Fig. 4b, which demonstrated that the A_1.0_-W film exhibits the transmittance modulation (ΔT) of 90.84% at 1100 nm. The transmittance spectral tests of the A_1.0_-W film grown for 10, 30, and 50 min are demonstrated as shown in Fig. 4c, which exhibit the ΔT of 44.86%, 90.84%, and 31.52%, at 1100 nm, respectively. Therefore, the A_1.0_-W films prepared with a AgNWs concentration of 1.0 mg mL^−1^ and the growth time of 30 min can achieve the best optical modulation performance.

The electrochromic cycling stability of the A_x_-W films is a critical parameter for evaluating the quality of electrochromic films. The transmittance curves of the A_1.0_-W, A_0.0_-W, A_0.5_-W, and A_1.5_-W films are measured at 1100 nm as shown in Figs. 4d and S17. The ΔT retention rate of the A_0.0_-W film after 300 cycles is only 57.38% (Fig. S17a), showing severe performance degradation. While the oxygen vacancies increase moderately and the initial conductive network is formed with the introduction of AgNWs enhancing the stability of the A_0.5_-W film keeping 68.40% modulation capability after 300 cycles. On the other hand, although the A_1.5_-W film exhibited relatively high stability keeping 81.0% modulation capability after 300 cycles, its initial ΔT was significantly reduced. It is notable that the A_1.0_-W film exhibits excellent cycling stability and can still maintain an initial ΔT of 86.37% after 20,000 cycles (Fig. 4d). Furthermore, the cyclic stability of the films in the VIS region also improves with the increase in the concentration of AgNWs (Fig. S18). As reported previously [54], the AgNWs form a conductive network within the film, facilitating rapid electron transport and reducing charge transfer resistance, thereby improving cycling stability. Additionally, the AgNWs effectively modify the interfaces between the FTO substrate and the A_1.0_-W film, enhancing adhesion and improving microstructural stability, which correlates with electrochemical cycling performance. Adhesion tests and SEM morphologies before and after cycling (Fig. S19) further confirm the strong adhesion of the A_1.0_-W film.

The bleaching (t_b_) and coloring (t_c_) times are as demonstrated in Fig. 4e. The response times of A_0.0_-W film were 3.2 and 7.4 s for t_b_ and t_c_, whereas the response times of A_1.0_-W film were 4.6 and 5.7 s for t_b_ and t_c_, respectively. Notably, despite distinct optical modulation ranges among the A_x_-W films, increasing AgNWs concentration consistently reduces response times. Color efficiency (CE) is also an important parameter, which quantifies the change in optical properties due to charge consumption per unit area. It is closely related to the energy consumption required for electrochromic films. This can be calculated using Eq. (7):

7 $$CE \, = \, {{\Delta OD} \mathord{\left/ {\vphantom {{\Delta OD} {\Delta Q_{d} }}} \right. \kern-0pt} {\Delta Q_{d} }} = \, \log \, {{\left( {T_{b} / \, T_{c} } \right)} \mathord{\left/ {\vphantom {{\left( {T_{b} / \, T_{c} } \right)} {\Delta Q_{d} }}} \right. \kern-0pt} {\Delta Q_{d} }}$$

where Δ*OD* represents the optical density, Δ*Q*_d_ denotes the per unit charge density, *T*_b_ and *T*_c_ represent the transmittance in the bleached and colored states at specific wavelengths, respectively. This study systematically examines the CE of A_x_-W films to elucidate the correlation between AgNWs concentration and electrochromic performance. As illustrated in Fig. 4f and Fig. S20, the measured CE values for A_0.0_-W, A_0.5_-W, A_1.0_-W, and A_1.5_-W films are 130.7, 91.0, 88.2, and 80.2 cm^2^ C^−1^ at 1100 nm, and 47.6, 37.2, 35.6, and 27.4 cm^2^ C^−1^ at 713 nm, respectively. This result revealed an inverse relationship between CE and AgNWs concentration. The AgNWs LSPR effect may partially obscure the intrinsic electrochromic contribution of WO_3_, consequently reducing the overall CE value.

Taking into account cyclic stability, response times and CE value collectively, the A_1.0_-W film demonstrates comprehensively superior performance, establishing it as the optimal sample in this study. To contextualize these results, we compared the A_1.0_-W film with WO_3_-based films reported in recent literature (Table S1). The scatter plot of ΔT versus cycling stability is shown in Fig. 4g, revealing that the A_1.0_-W film not only achieves the highest ΔT but also exhibits leading cycling stability. Similarly, Fig. 4h displays the relationship between ΔT and total response time (t_b_ and t_c_), where the A_1.0_-W film maintains a high ΔT while simultaneously delivering relatively fast switching speeds. These comparative analyses further validate the exceptional overall performance of the A_1.0_-W film across all critical electrochromic metrics.

The A_1.0_-W film was further tested through a sunlight radiation simulation experiment. As shown in Fig. 4i, the sunlight source passed through the A_1.0_-W film and shone on the black fabric. The temperature of the black fabric after being irradiated for 2 min was detected by a temperature thermal imager. It was found that the temperature of the fabric at the bottom of the traditional glass was 47.4 °C, and the temperatures of the A_1.0_-W film in the bleached and colored states were 45.3 and 42.5 °C, respectively. The heat insulation capacity of the A_1.0_-W film in the bleached state is comparable to that of ordinary glass, while the film in the colored state has a certain heat insulation capacity. These experiments verified the infrared radiation heat management capability of A_1.0_-W film in the application of smart windows, providing a reference for the design of energy-saving building materials.

### Regulation Assessment of ESWs

**Fig. 5:**
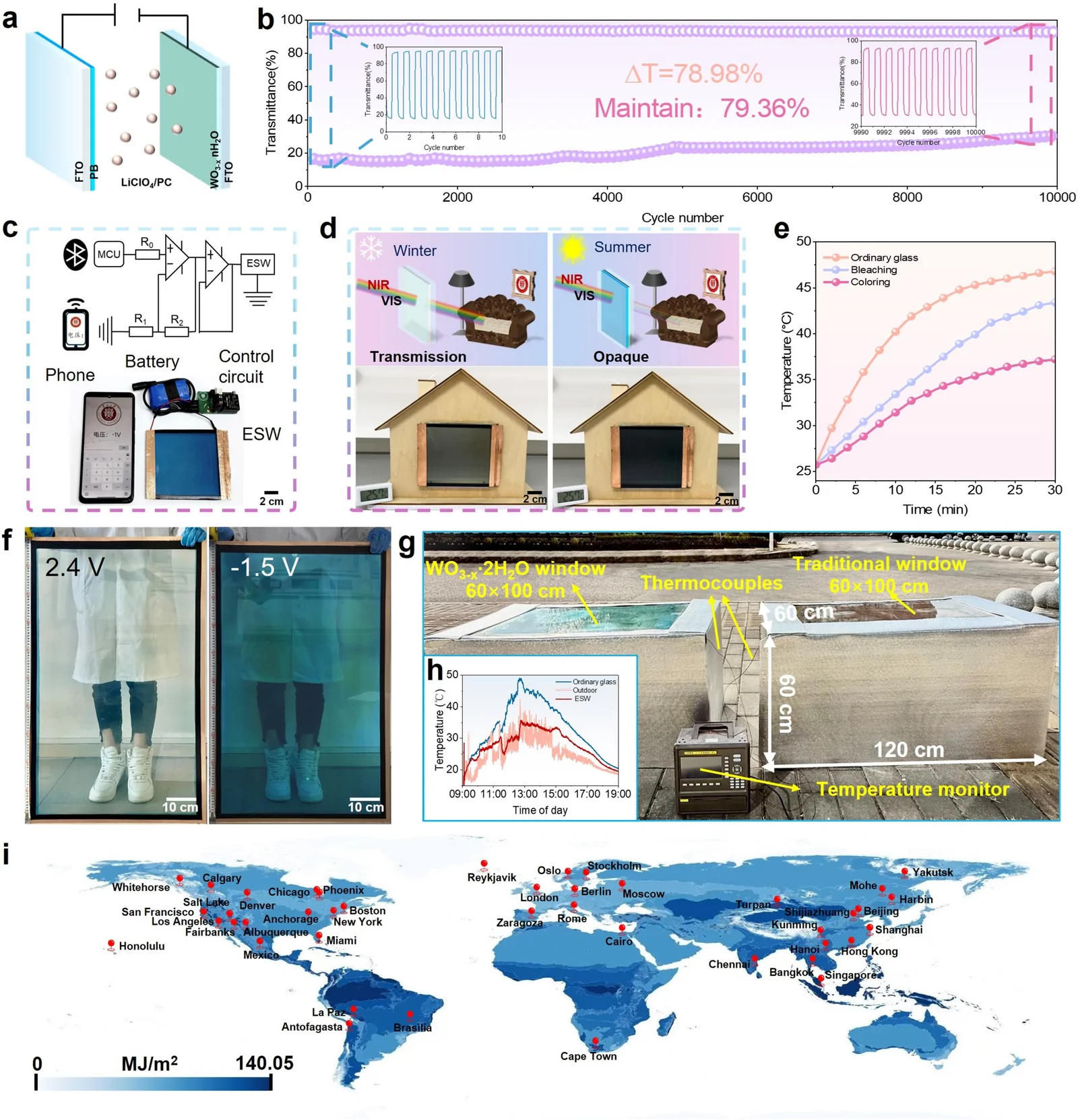
**a** Schematic diagram of the device. **b** Graph of the number of cycles of the device at 1100 nm. **c** Control circuit of the ESW and its display photographs. **d** Main application scenarios of ESW and its control demonstration. **e** Internal temperature of the model variation over time. **f** Large-scale ESW photographs at 2.4 V (bleaching) and -1.5 V (coloring). **g** Schematic illustration of the outdoor measurement setup. **h** Outdoor test temperature–time curve graph. **i** Global energy-saving map of the building model with electrochromic smart windows

Given the outstanding performance of the A_1.0_-W film, it was assembled into electrochromic devices (ECDs) of three different sizes as smart window samples to evaluate their overall performance. The ECDs were assembled with the layered structure as illustrated in Fig. 5a. The preliminary 2 × 4 cm^2^  ECD was fabricated to evaluate fundamental electrochromic characteristics. As demonstrated in Fig. S22, the ECD showed substantial optical modulation when switching between −1.2 V (colored state) and + 2.4 V (bleached state), achieving exceptional ΔT values of 75.1% at 713 nm and 78.9% at 1100 nm. These results confirmed the effective dual-band modulation capability of ECD across both VIS and NIR spectral regions, which was further verified by voltage-dependent transmittance spectra (Fig. S23). Notably, the device exhibited particularly impressive performance at 1100 nm, with high CE value of 235.5 cm^2^ C^−1^ and rapid switching times of 7.6 s (bleaching) and 9.6 s (coloring), as shown in Fig. S24. Comparable performance was observed at 713 nm, where the device achieved a CE value of 130.4 cm^2^ C^−1^ along with even faster switching times of 4.0 s (bleaching) and 2.1 s (coloring) (Fig. S25). The ECD exhibited outstanding cycling stability, a crucial parameter for practical electrochromic applications. Accelerated durability testing revealed excellent performance retention at both NIR and VIS wavelengths. At 1100 nm, the device maintained 80% of its initial ΔT after 10,000 cycles (Fig. 5b). Superior stability was also achieved at 713 nm, and ΔT showed virtually no attention after 7000 cycles. (Fig. S26). These results demonstrate the excellent electrochromic performance across both spectral ranges for ECD based A_1.0_-W film. To further verify the stability of the ECD, an accelerated aging test was conducted using a Programmable Xenon Lamp Weathering Tester, with the corresponding test conditions provided in the Supporting Information. The full transmittance spectra and digital photographs of the ECD before and after aging are shown in Fig. S27. The full transmittance spectra before and after aging show that the transmittance only decreased by 2.3% in the NIR (1100 nm) and 1.4% in the VIS (713 nm). The ECD still maintains an excellent optical modulation range, demonstrating outstanding cycling stability and anti-aging performance.

Furthermore, a 10 × 10 cm^2^  ECD was fabricated and integrated with a custom-designed control system for wireless and intelligent operation. The circuit schematic and system architecture are depicted in Fig. 5c. The system established Bluetooth Low Energy communication with a smartphone APP, enabling users to precisely set the target voltage via the app interface. The input signal is transmitted to a microcontroller unit (MCU), which subsequently generates the corresponding voltage output. This signal is then amplified by an operational amplifier and a power amplifier before being applied to the ECD, facilitating reversible and controllable color switching. A real-time demonstration of the dynamic optical transition is provided in Movie S1. The practical implementation of the ESW is illustrated in Fig. 5d. Additionally, Movie S2 showcases the user-friendly smartphone app interface, allowing seamless switching between different ESW operating modes. This intuitive and user-friendly operation method enables seasonal adaptive and human intention control of ESW states, optimizing both indoor thermal comfort and visual suitability. Such programmable functionality highlights the system potential for seamless integration into smart buildings and underscores its viability for large-scale commercial deployment in sustainable architecture.

To evaluate the thermal regulation performance of warm and cold modes compared to traditional windows, a comparative experiment using a standard glass panel as a control under simulated solar irradiation was conducted (Fig. S28). The temperature evolution curves, recorded at an initial room temperature of 25.7 °C, demonstrated distinct thermal behaviors (Fig. 5e). The cold, warm, and traditional glass modes exhibited peak temperatures of 37.2, 43.4, and 46.8 °C, respectively. Notably, the thermal response of warm mode closely mirrored that of conventional windows, confirming that the ESW in its bleached state maintains comparable thermal properties to standard glazing. In contrast, the cold mode significantly attenuated temperature rising of 9.6 °C compared traditional glass experiment, demonstrating the effective radiative cooling capability of ESW in the colored state. To further quantify the heat-blocking performance, an additional solar radiation simulation experiments were performed by measuring the temperature of black fabric exposed to transmitted sunlight (Fig. S29). After 2 min of solar irradiation, thermal imaging revealed substrate temperatures of 45.4, 43.0, and 35.3 °C for traditional glass and ESW in bleached and colored state, respectively. The significant difference confirmed that the bleached state ESW exhibits near-equivalent thermal transmission to traditional glass. The colored state ESW provides substantial heat insulation capacity as 10.1 °C reduction compared with traditional glass. Collectively, these experiments validated dynamic thermal management capacity of the ESW, highlighting its dual-mode functionality for smart window applications.

Although the A_1.0_-W film-based ECD demonstrated exceptional electrochromic performance and effective solar radiation thermal management, large-scale ESW fabrication remains the primary challenge for commercial adoption. In this study, we successfully fabricated a uniform A_1.0_-W film with dimensions of 100 × 60 cm^2^ and assembled it into a functional large-area ESW, as shown in Fig. 5f. The large-scale ESW demonstrated reversible transitions between two well-defined states, a highly transparent state at + 2.4 V and a uniformly deep-blue-colored state at −.5 V. To assess optical uniformity, the nineteen discrete points were selected to spectral measurements in the large-area ESW. The results showed relatively consistent L*, a*, and b* values across all measurement locations (Fig. S31), confirming excellent switching homogeneity uniformity. Furthermore, cyclic stability tests were conducted on the large-area ESW using an electrochemical workstation and a colorimeter (Fig. S32). It was found that the large-area ESW remained stable after 500 cycles, indicating its excellent cyclic stability performance. Meanwhile, the changes in response time at the edge and center of the large-area ESW were also tested, as illustrated in Fig. S33.

To evaluate the thermal regulation performance of the large-area ESW based on the A_1.0_-W film in real ambient conditions, two identical house models were installed with ordinary glass and ESW (Fig. 5g) and tested under natural sunlight in Guiyang, China (26.11°N, 106.07°E; 1659 m altitude). Temperature fluctuations within both the interior and exterior of these model houses were recorded using a thermocouple from 9 a.m. to 7 p.m. on November 16, 2025. As shown in Fig. 5h, when the ESW is in the colored state, it achieves a temperature regulation of approximately 9 °C at noon compared with traditional glass, demonstrating excellent thermal management capabilities. These characteristics satisfy the operational requirements for practical applications. The successful fabrication and performance validation of this large-area ESW mark a significant advancement toward commercialization. Unlike many existing studies (Table S2), this work provided a comprehensive evaluation encompassing film properties, device performance, and scalability. The developed ESW exhibited superior characteristics in exceptional optical modulation capability, remarkable cycling stability, and large-area manufacturability. However, further research is still needed regarding the weather resistance of large-area ESW under harsh conditions and extreme environments.

To systematically evaluate the energy-saving potential of the ESW, comprehensive building energy simulations were performed using EnergyPlus software (Fig. S34, model specifications detailed in Table S3). The study encompassed over 40 global locations representing diverse Köppen–Geiger climate classifications (see Supporting Information for complete methodology) [55]. Comparative analysis revealed that ESWs significantly outperform traditional glass in energy efficiency across all evaluated climates (Fig. S35). The most pronounced savings were observed in tropical regions, 140.0 MJ m^−2^ in Chennai, 137.9 MJ m^−2^ in Bangkok, 138.4 MJ m^−2^ in Singapore. The comprehensive visualization of energy-saving performance of the ESW across global climate zones is presented in Fig. 5i, demonstrating its substantial advantages over traditional glazing systems. These results highlight the ESW exceptional potential for reducing building energy consumption worldwide. In addition, to evaluate the reliability of ESW in practical architectural applications, an impact test was conducted on ESW and glass of the same thickness (8 mm), with the relevant test methods provided in the Supporting Information. As observed in the comparative Fig. S36 before and after the test, compared with traditional glass, ESW showed no obvious cracks after impact, while traditional glass developed numerous cracks and shattered completely upon impact. Thus, it is concluded that ESW exhibits high reliability in architectural applications.

## Conclusion

In conclusion, the A_x_-W films were successfully prepared by an economical, environmentally friendly, efficient, practical, reliable, and simple immersion process. Combined with the strategy of AgNWs modification, which utilized sacrificial reducing agents to introduce oxygen vacancies and promote the growth of the films, the growth process and enhancement mechanism of A_x_-W films were clarified through aspects such as morphology, oxygen vacancy concentration, DFT theoretical calculation, and electrochemical characteristics. The A_1.0_-W film has high optical modulation (90.8% at 1100 nm) and excellent cycling stability (with a retention rate of 86.37% after 20,000 cycles). The ESW based on A_1.0_-W film also exhibited excellent electrochromic performance, including impressive transmittance modulation (75.1% at 713 nm and 78.9% at 1100 nm), high CE value (130.4 cm^2^ C^−1^ at 713 nm and 235.5 cm^2^ C^−1^ at 1100 nm), fast switching time (t_c_ = 2.1 s, t_b_ = 4 s at 713 nm and t_c_ = 9.6 s, t_b_ = 7.6 s at 1100 nm), and exceptional cyclic stability (no attention after 7000 cycles at 713 nm and 80.0% after 10,000 cycles at 1100 nm). Meanwhile, due to the simple preparation process of simple immersion process, large-area A_1.0_-W film and ESW (100 × 60 cm^2^) were effortlessly fabricated. Additionally, cyclic stability tests, outdoor thermal management tests, anti-aging tests, energy-saving simulation tests, and impact resistance tests were conducted on the ESW. These results demonstrate that the ESW possesses excellent durability, high energy-saving efficiency, and practicality. This approach provides a new route for the design of ESW and is of great significance for exploring advanced energy-saving devices with highly selective solar radiation regulation.

## Supplementary Information

Below is the link to the electronic supplementary material.

Supplementary file1 (MP4 4445 KB)

Supplementary file2 (MP4 6118 KB)

Supplementary file3 (DOCX 21186 KB)
