# Supplementary material for: Scalable Fabrication of Large-Scale Electrochromic Smart Windows for Superior Solar Radiation Regulation and Energy Savings
Source: Nanomicro Lett. 2026 Jan 16;18:211. doi: 10.1007/s40820-025-02055-x (PMC12811191; doi:10.1007/s40820-025-02055-x)
Supplement: Supplementary file 3 — Supplementary file3 (DOCX 21186 KB) [file 40820_2025_2055_MOESM3_ESM.docx]

Supporting Information for

**Scalable Fabrication of Large-Scale Electrochromic Smart Windows for Superior Solar Radiation Regulation and Energy Savings**

Yanbang Tang^1^, Junyu Yuan^2,3^, Rongzong Zheng^1,2^*, Chunyang Jia^2,3^*

^1^College of Materials and Metallurgy, Guizhou University, Guiyang 550025, P. R. China

^2^State Key Laboratory of Electronic Thin Films and Integrated Devices, National Engineering Research Center of Electromagnetic Radiation Control Materials, School of Integrated Circuit Science and Engineering, University of Electronic Science and Technology of China, Chengdu 610054, P. R. China

^3^Shenzhen Institute for Advanced Study, University of Electronic Science and Technology of China, Shenzhen 518110, P. R. China

*Corresponding authors. E-mail: [rzzheng@gzu.edu.cn](mailto:rzzheng@gzu.edu.cn) (Rongzong Zheng); [cyjia@uestc.edu.cn](mailto:cyjia@uestc.edu.cn) (Chunyang Jia)

Text S1 Theoretical calculations

All the density-functional theory (DFT) computations were performed using the Cambridge Sequential Total Energy Package (CASTEP) based on the pseudopotential plane wave (PPW) method [S1]. Electron-ion interactions were described using the ultrasoft (USP) potentials [S2]. A plane-wave basis set was employed to expand the wave functions with a cutoff kinetic energy of 400 eV. For the electron-electron exchange and correlation interactions, the functional parametrized by Perdew-Burke-Ernzerhof (PBE) [S3], a form of the general gradient approximation (GGA), was used throughout. The vander Waals interaction was described using the DFT-D2 method that proposed by Grimme [S4].

During the geometry optimizations, all the atom position were allowed to relax. In this work, the Brillouin-zone integrations were conducted using Monkhorst-Pack (MP) grids of special points with the separation of 0.04 Å^-1^ for the model cell [S5]. The convergence criterion for the electronic self-consistent field (SCF) loop was set to 1×10^-6^ eV/atom. The atomic structures were optimized until the residual forces were below 0.03 eVÅ^-1^.

Text S2 Measurements and characterizations

The morphology of the A_x_-W films was characterized by SEM (ZEISS Gemini 300). The elemental valence states of the A_x_-W films were characterized by XPS (Thermo Scientific K-Alpha). The phase and structure of the A_x_-W films were characterized by an X-ray diffractometer (XRD, D8 Advance, Bruker, Germany) and a transmission electron microscope (TEM, F200, Japan). Raman spectra with an excitation wavelength of 532 nm were recorded at room temperature using a surface-enhanced Raman spectrometer (ATR6500), with a wavenumber range of 200-4000 cm^-1^. Electrochemical experiments were conducted using a three-electrode system electrochemical workstation (CHI 760 E, Shanghai Chenhua), and ultraviolet transmission and absorbance curves were measured by S6300 (Shanghai Mapuda). In-situ color coordinates were recorded by a spectrophotometer (YS 5410, Shenzhen 3NH). Programmable Xenon Lamp Weathering Tester.

Electrochemical tests were carried out using a three-electrode electrochemical cell. The reference electrode was Ag/AgCl, the counter electrode was a platinum sheet, and the working electrode was an A_x_-W film coated on FTO. Cyclic voltammetry (CV) measurements of the A_x_-W films were conducted between -1.0 and 1.0 V at a scan rate of 50 mV s^-1^. The electrochemical impedance spectroscopy (EIS) was conducted over a frequency range of 10,000 to 0.1 Hz with a voltage of -0.5 V and an amplitude of 10 mV. Quantitative impedance analysis was performed using ZSimpwin software with the equivalent circuit. The results indicate that the R_ct_ values for A_0.0_-W, A_0.5_-W, A_1.0_-W and A_1.5_-W films are 35.5, 33.6, 30.4 and 28.3 Ω, respectively, while the Z_w_ values are 5.3×10^-2^, 5.7×10^-2^, 5.7×10^-2^ and 5.8×10^-2^ Ω cm^-2^, respectively. The current density-voltage (I-V) curves of A_x_-W films measured within -1.5 V to 1.5 V at a scan rate of 100 mV/s. Multi-potential step tests were performed on the A_x_-W films under two-step cycles of -1 V/20 s and 1 V/20 s. For electrochromic device (ECD), it was set as 2.4 V/15 s and -1.2 V/15 s at 1100 nm, and 2.4 V/25 s and -1.2 V/20 s at 713 nm.

Text S3 Electrochromic smart window (ESW) control system integration

The circuit schematic diagram of the ESW intelligent control system is shown in Fig. 5c. The output voltage range of the DAC of the microcontroller (MCU) is 0 to 2 V. After applying the bias, this voltage is converted to V_in_, which ranges from -1 V to +1 V. V_in_ is then amplified by a non-inverting amplifier and a unity-gain buffer to produce an output voltage of ±3 V. The specific relationship is as follows:

V_out_ = V_in_ (R_1_ + R_2_) / R_1_

Here, V_in_ is the biased voltage, and V_out_ is the output voltage. R_0_, R_1_, and R_2_ are resistors with values of 10 kΩ, 10 kΩ, and 20 kΩ, respectively.

Text S4 Thermal insulation performance of the A_1.0_-W film and the ESW

The study achieved two states of bleaching and coloring by applying different voltages (2.4 V and -1.2 V) to ESW, corresponding to warm and cold modes, respectively, as shown in Fig. 5d. To compare the effects of these two modes and conventional windows on room temperature, a common glass was used to simulate a conventional window. All windows were tested at a room temperature of 24.2°C and then exposed to a lamp simulating sunlight (Fig. S28). Temperature changes were recorded every two minutes within 30 minutes.

Text S5 Sunlight radiation simulation of the A_1.0_-W film and the ESW

The sunlight source passed through the A_1.0_-W film and shone on the black fabric. The temperature of the black fabric after being irradiated for 2 minutes was detected by a temperature thermal imager, as shown in Fig. 4i.

The sunlight source passed through the ESW and shone on the black fabric. The temperature of the black fabric after being irradiated for 2 minutes was detected by a temperature thermal imager (Fig. S29).

Text S6 ECD aging resistance test

An accelerated aging test was conducted in accordance with GB/T 16422.2-2022 using a microcomputer-controlled xenon lamp aging tester. The test conditions were set as follows: temperature controlled at 65°C, humidity at 55%, and irradiance at 0.51 W/(m^2^·nm). A single aging cycle lasted 2 hours, consisting of 1.42 hours of irradiation followed by 0.18 hours of water spray, with the cycles repeated consecutively for a total duration of 100 hours.

Text S7 Energy-saving performance simulation

The energy costs of the building were simulated using EnergyPlus 23.2.0 and Open Studio 2021 software. In the simulation, the building has dimensions of 27 m (length) × 18 m (width) × 7 m (height). The north axis of the building is at a 0-degree angle to true north. The compositions of the roof, floor and walls are shown in Table S3. The window-to-wall ratio and windowsill height are set to 0.28 and 0.9 m, respectively. The cooling and heating sources are provided by an ideal load air system. The thermostat setpoints are 25°C for cooling and 15°C for heating. The energy-saving performance of the building is benchmarked against 3 mm transparent glass. The energy consumption of the windows in different states is calculated hourly, and then the minimum values are summed to obtain the annual minimum ideal energy consumption. The weather files contain typical hourly weather data, which can be downloaded from the official EnergyPlus Weather Data website. Python was used to plot the energy-saving diagrams. Considering data availability and completeness, 33 cities located in different climate zones were selected, and it is assumed that the weather files of these cities correspond to the entire climate zone. The climate zone classifications and representative city data are listed in Table S4.

Text S8 Cost-effectiveness of the large-scale WO_3_-based ESWs

This study successfully fabricated large-area WO_3_-based ESWs. Evaluating the cost-effectiveness is crucial for determining the feasibility of practical applications. This device adopts a simple immersion process and packaging process, which not only has an efficient manufacturing process but also has a low material cost, while ensuring stable product quality control. Based on these advantages, large-area WO_3_-based ESWs demonstrate outstanding economic feasibility and practical potential in large-scale industrial applications.

1. Material costs

The large-area WO_3_-based ESWs fabricated in this study consist of the following materials: FTO conductive glass, WO_3_ film, an electrolyte layer, and Prussian blue (PB) film. The estimated material costs are detailed below:

(1) FTO glass: FTO glass serves as a transparent conductive substrate crucial for electrochromic performance. Estimated cost: ~$110-$120 m^-2^.

(2) WO_3_ film and PB film: Estimated cost: The cost for laboratory preparation is approximately $25-$30 0.6 m^-2^.

(3) LiClO_4_/PC electrolyte: LiClO_4_: ~$0.28 gram^-1^, PC: ~$0.1 milliliter^-1^.

2. Manufacturing costs

The container for pre-prepared precursor solution: Estimated cost: ~$15.8.

The pool：Estimated cost: ~$7.2.

The calorifier：Estimated cost: ~$3.7.

These devices can all be reused (Fig. S29).

3. Durability

Durability serves as a critical determinant for evaluating the cost-effectiveness of ECD. Experimental results demonstrate that WO_3_ films maintain excellent stability over 20,000 cycles with a 13% decay, while the large-area WO_3_-based ESWs exhibit remarkable cycling stability over 10,000 cycles with only 15% degradation. Assuming an annual operation frequency of 200 switching cycles, this cycling endurance translates to a service life of 50 years. Based on the aforementioned excellent cycling stability, the large-scale WO_3_-based ESWs demonstrate great potential for application in areas such as smart windows, energy-efficient buildings, and dynamic displays. Furthermore, integration with artificial intelligence or adaptive control strategies could enable more intelligent and efficient electrochromic systems, thereby expanding its applications in sustainable energy and green building technologies.

4. Energy savings evaluation

To systematically evaluate the energy-saving potential of the ESW, comprehensive building energy simulations were performed using EnergyPlus software. Comparative analysis revealed that ESWs significantly outperform traditional glass in energy efficiency across all evaluated climates (Fig. S30). The most pronounced savings were observed in tropical regions, 140.0 MJ m^-2^ in Chennai, 137.9 MJ m^-2^ in Bangkok, 138.4 MJ m^-2^ in Singapore.

1 kwh = 3.6 MJ

These results highlight the ESW exceptional potential for reducing building energy consumption worldwide.

Text S9 Preparation of large-area electrochromic smart windows

(1) **Preparation of Prussian** b**lue** (**PB**) **precursor solution:** Initially, an amount of 0.062 g FeCl_3_, 0.146 g K_3_Fe(CN)_6_, and 0.5445 g KCl was dissolved in 100 mL of deionized water, resulting in the formation of a Prussian blue solution (FeHCF). Subsequent to this, a quantity of 0.095 g of K_2_MoO_4_, 0.124 g of K_3_Fe(CN)_6_, and 2.928 g of KCl was weighed and dissolved in 25 mL of deionized water, resulting in the generation of a pale yellow, clarified solution of a molybdenum based Prussian blue analogue (MoOHCF). The two distinct solutions were then combined in a ratio of 4:1 and subjected to sonication using an ultrasonic cleaner until no signs of precipitation were observed. This process produces the desired precursor solution of FeHCF/MoOHCF.

MXene was coated onto the FTO substrate by spraying or spin-coating. After drying in an oven for 5 minutes, the FTO glass was placed in the prepared precursor solution. After 15 minutes of immersion at 45°C, the homogeneous PB films were produced [S6].

(2) **Preparation of large-area WO_3_ film:** Weigh 0.3 g of Na_2_WO_4_·2H_2_O and dissolve it in 30 mL of deionized water. Subsequently, 0.6 g of citric acid is added to the solution and ensure complete dissolution. Finally, 3.5 mL of 3 M HNO_3_ was added. The resulting mixture is stirred for 20 min to obtain a WO_3_ precursor solution.

The spry-coating the AgNWs ethanol suspension (1.0 mg/mL) onto the FTO substrate (0.04 mL/cm^2^), dried in air atmosphere for 30 min. Then, pour a certain amount of WO_3_ precursor solution into a reaction tank (Fig. S30) and immerse the dry AgNWs/FTO substrate in the precursor solution. Heat in a 60°C water bath for 30 min, remove FTO for rinsing, and remove the sediment on the back. After drying, the large-area WO_3_ films are obtained.

Text S10 ESW Impact Test

Select a piece of glass with the same thickness as ESW (8mm). Under normal temperature conditions, keep the impact surface horizontal. In accordance with GB/T 15763.2-2005, a smooth steel ball with a diameter of 63.5 mm (approximately 1000 g) is placed at a height of 100 cm above the sample surface and allowed to fall freely, with the impact point within 25 mm of the sample center. The purpose is to observe whether the sample is damaged.

**Supplementary Figures**


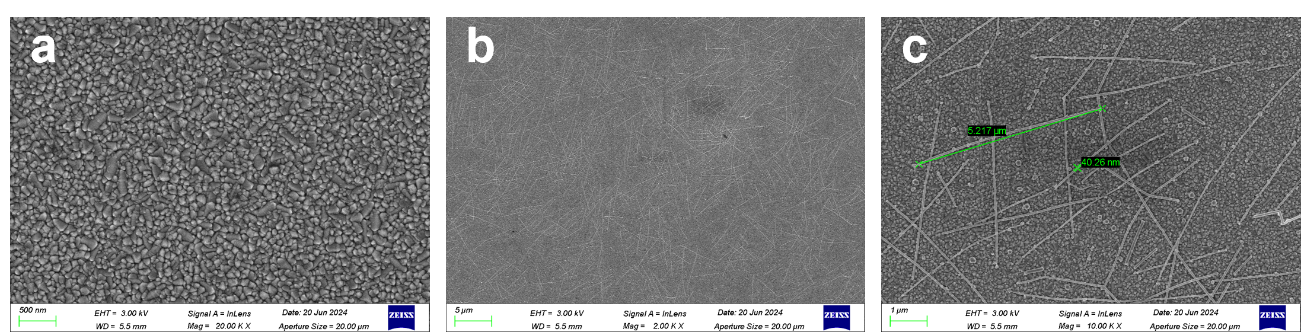


Fig. S1 SEM characterization of FTO substrate and AgNWs. (a) FTO substrate. (b-c) Spin coating of AgNWs on FTO substrate


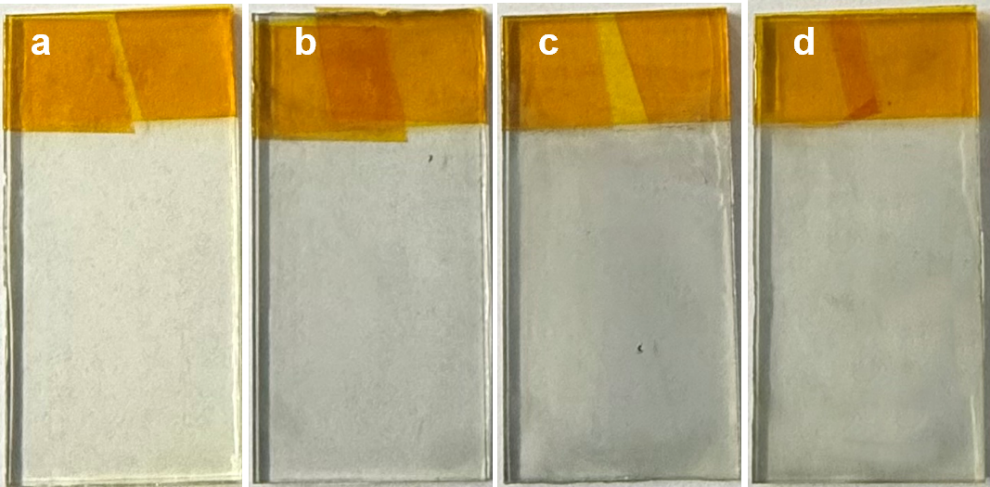


Fig. S2 Digital photos of the A_x_-W films prepared with different concentrations of AgNWs. (a) A_0.0_-W film. (b) A_0.5_-W film. (c) A_1.0_-W film. (d) A_1.5_-W film


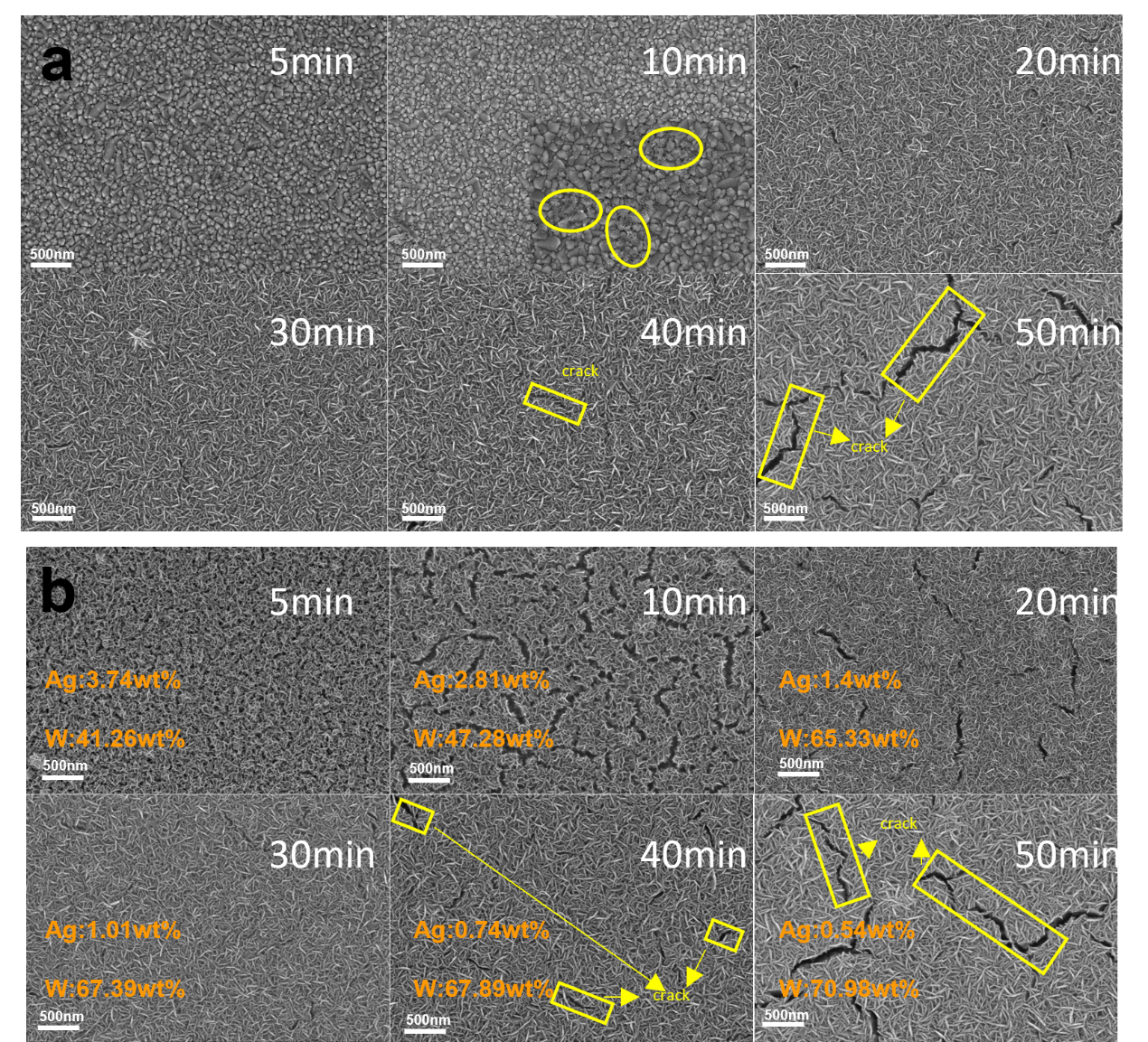


Fig. S3 SEM morphological images of the A_0.0_-W film and A_1.0_-W film at different growth times. (a) A_0.0_-W film. (b) A_1.0_-W film


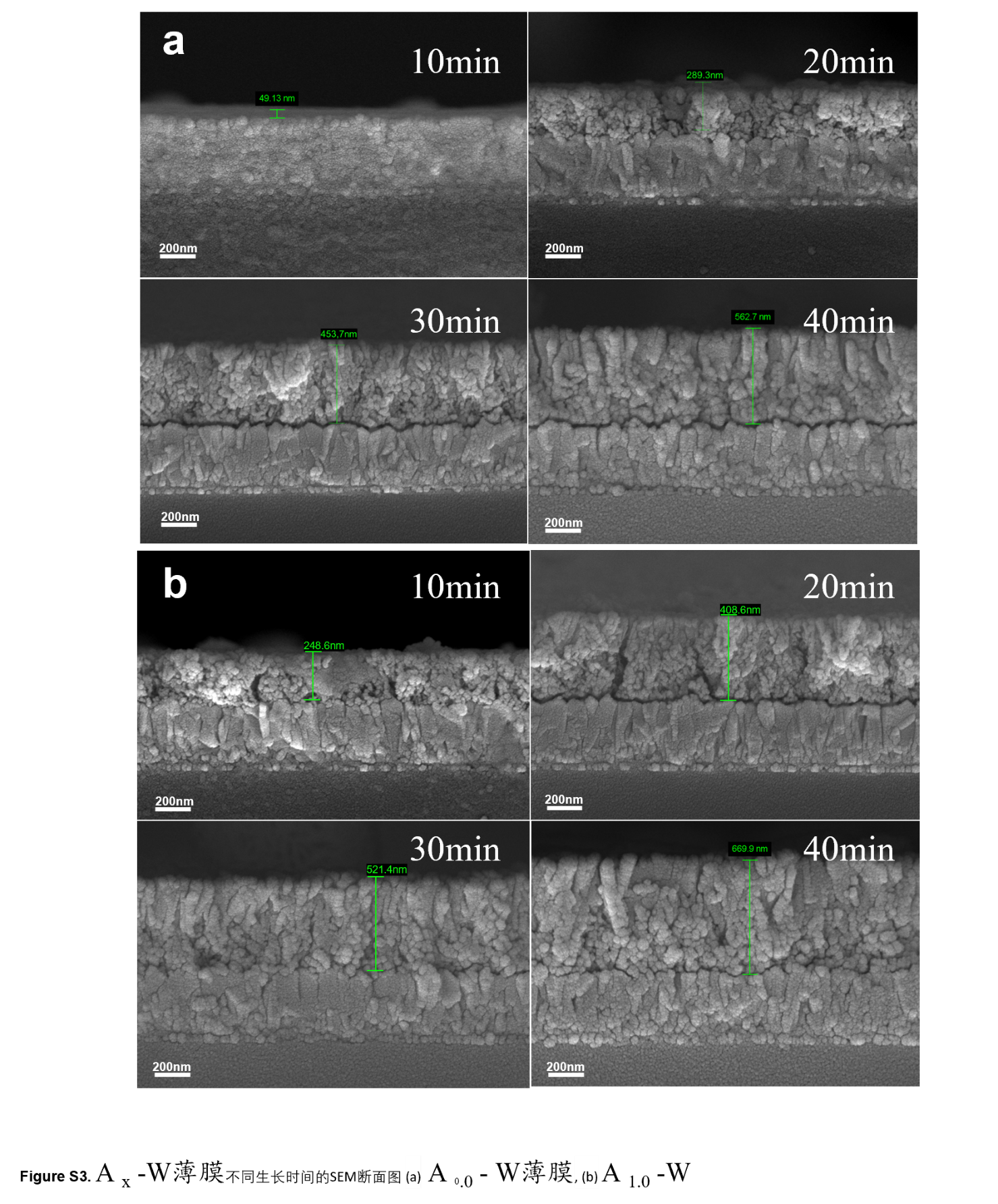


Fig. S4 SEM morphological images of the A_0.0_-W film and A_1.0_-W film at different growth times. (a) A_0.0_-W film. (b) A_1.0_-W film


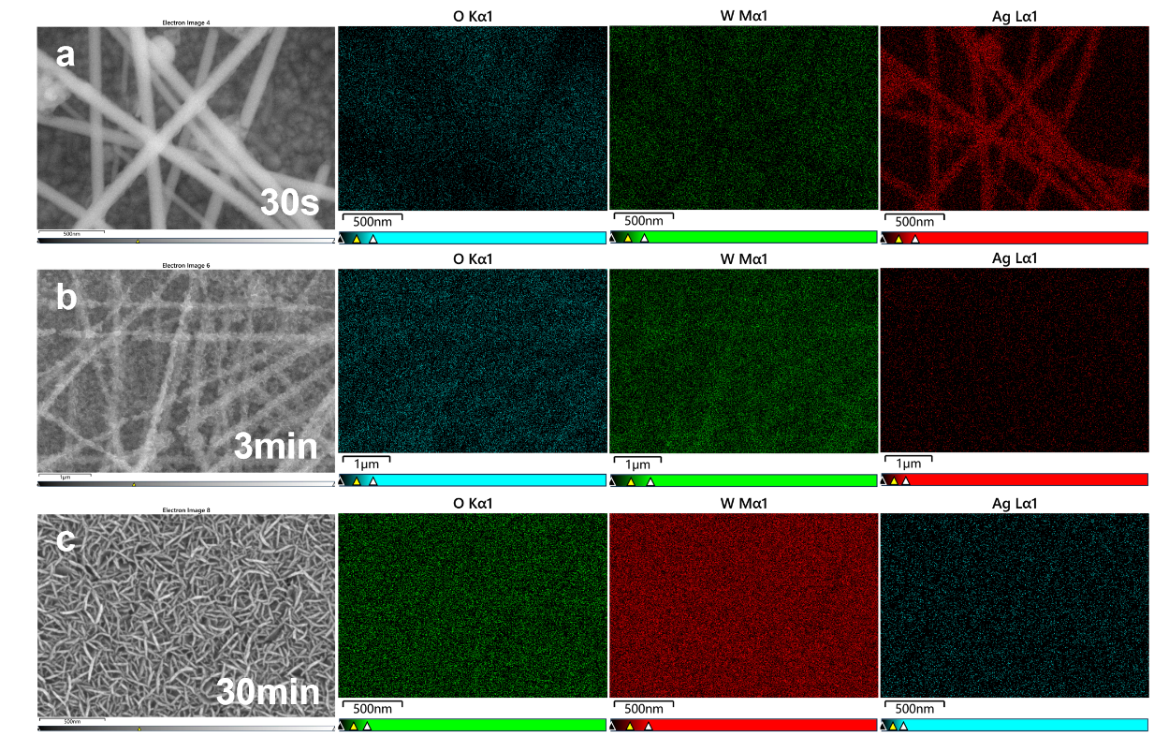


Fig. S5 EDS images of A_1.0_-W film with different growth times. (a) 30s. (b) 3min. (c) 30min

**
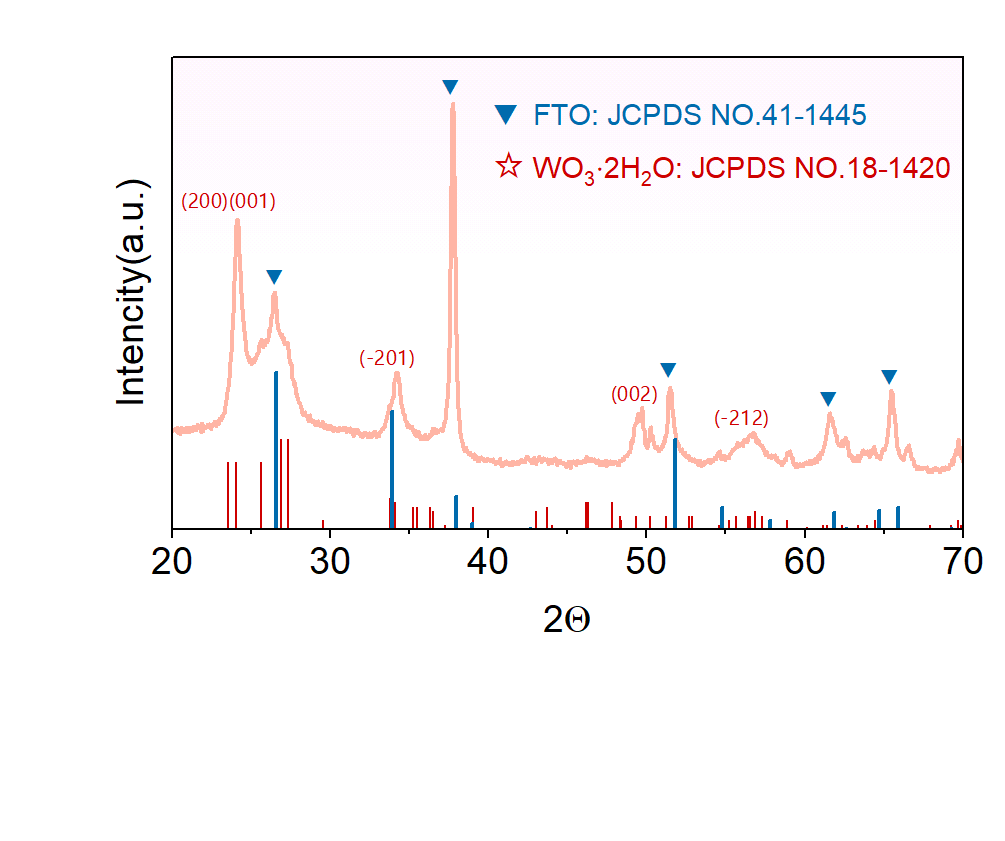
**

**Fig. S6** XRD pattern of A_1.0_-W film


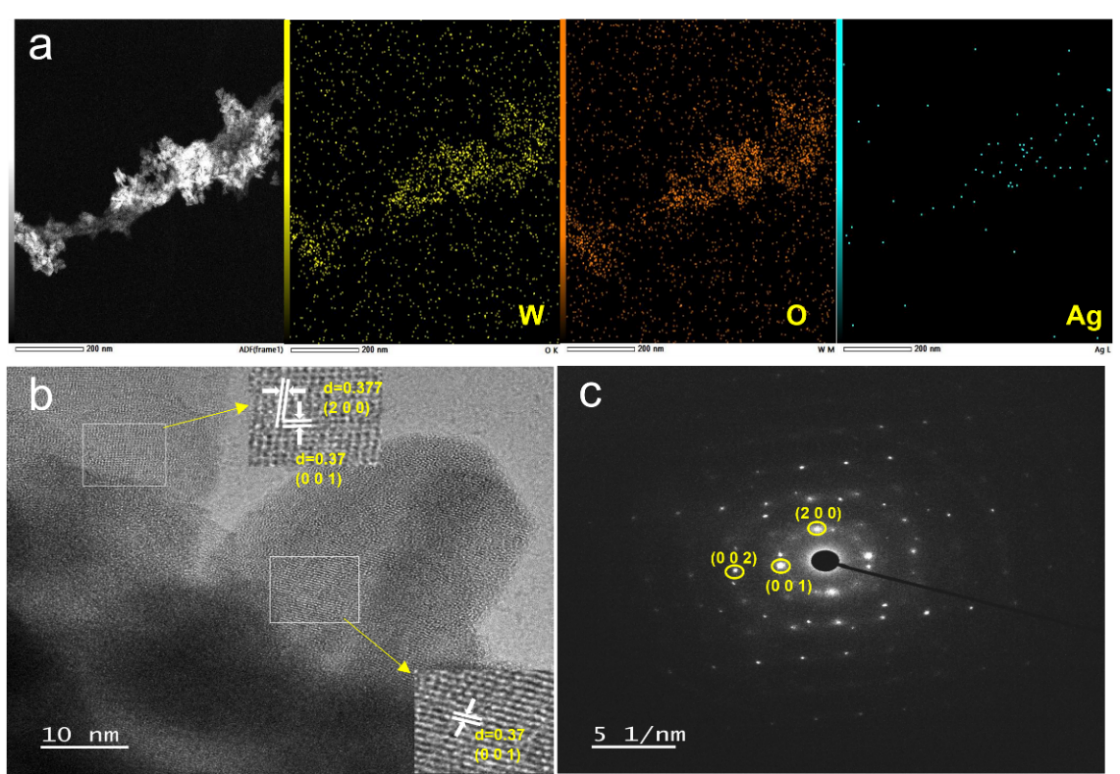


Fig. S7 The EDS, TEM and SEAD plots of A_1.0_-W film. (a) EDS plot. (b) TEM plot. (c) SEAD plot


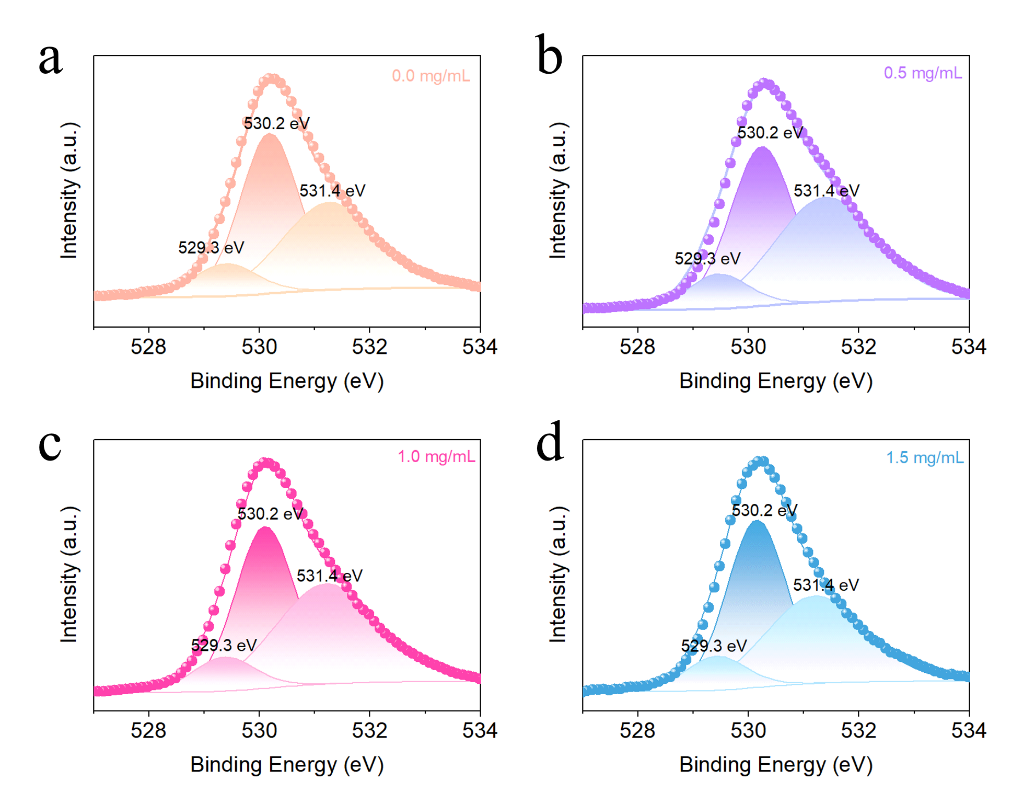


Fig. S8 The O1s XPS profile in different AgNWs concentration of A_x_-W films. (a) 0.0 mg/mL. (b) 0.5 mg/mL. (c) 1.0 mg/mL. (d) 1.5 mg/mL

**
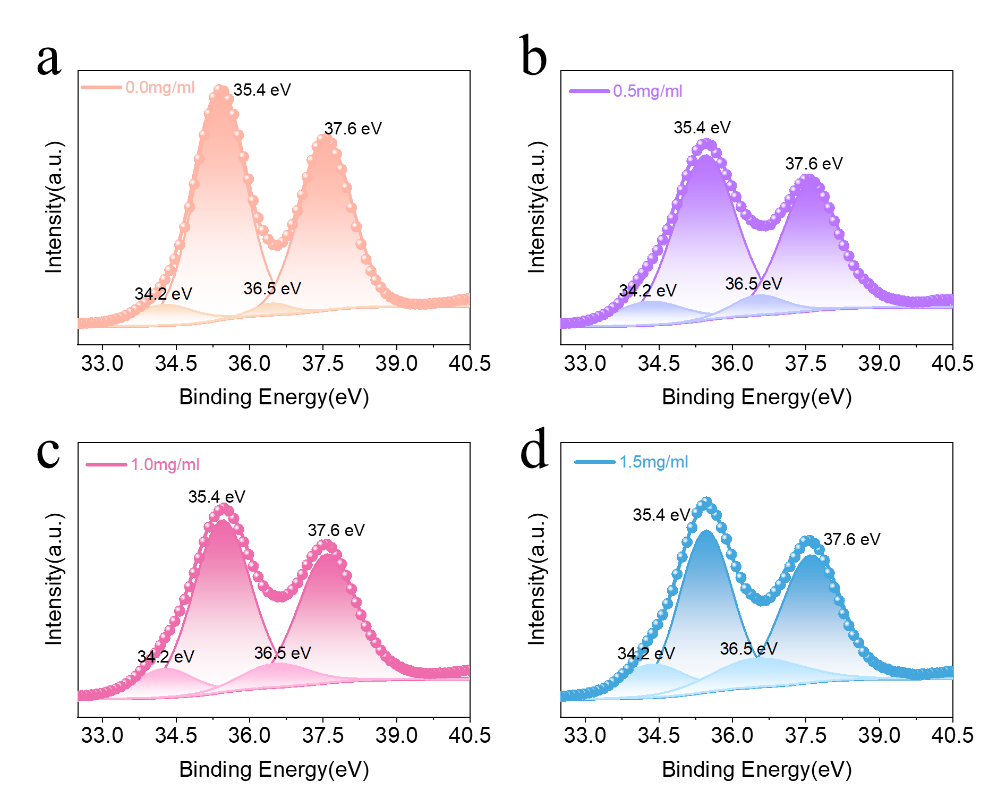
**

Fig. S9 W4f spectrum of A_x_-W film


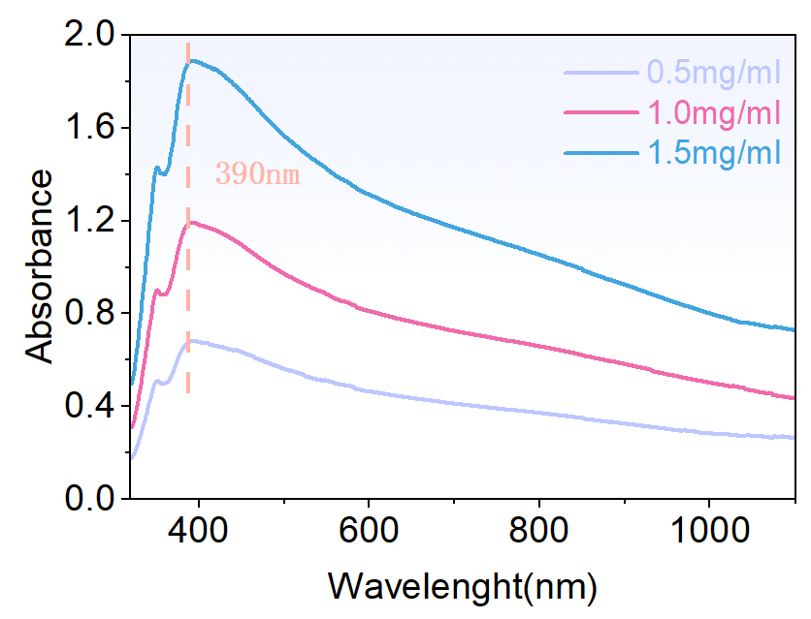


Fig. S10 Absorption spectra of different concentrations of AgNWs


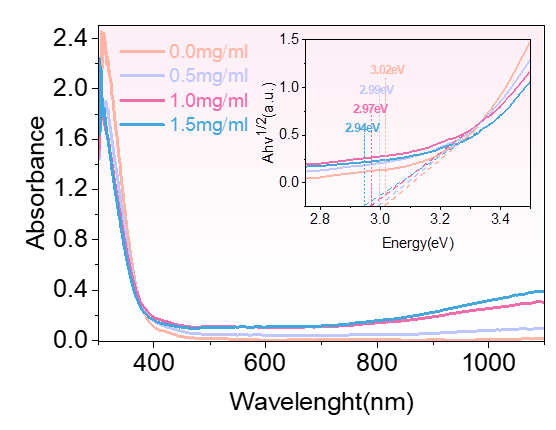


Fig. S11 Absorption spectrum and Tauc pattern of A_x_-W films


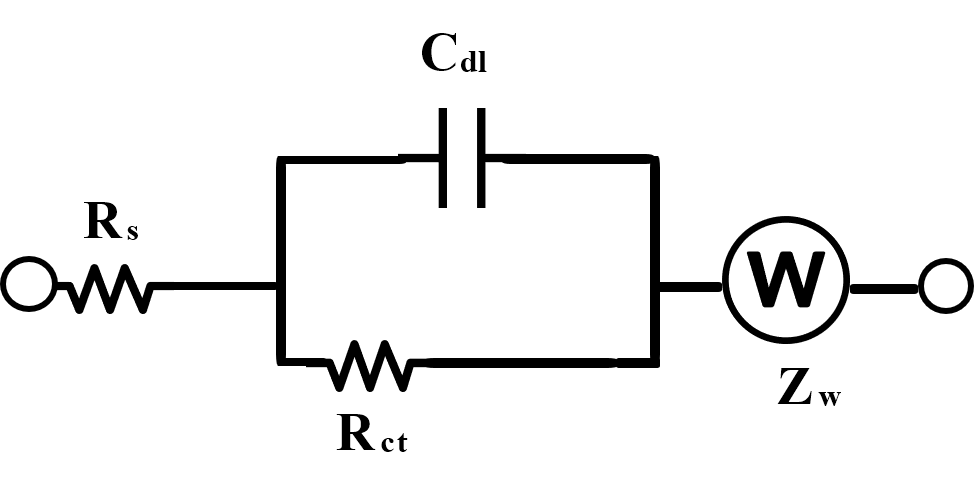


Fig. S12 Equivalent circuit model of an A_x_-W films

**
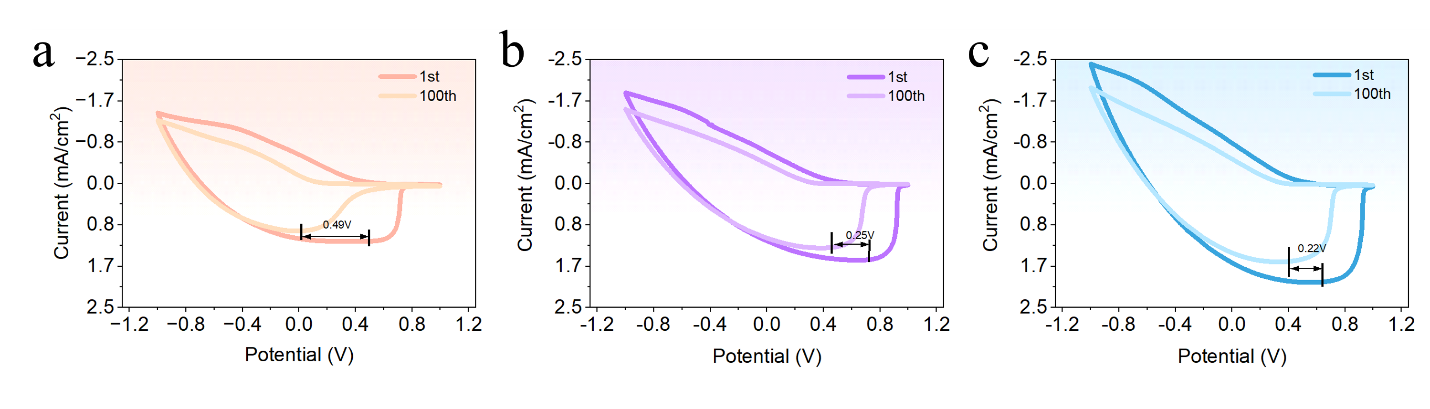
**

Fig. S13 100-turn CV cycle test plot of A_x_-W films. (a) A_0.0_-W film. (b) A_0.5_-W film. (c) A_1.5_-W film.


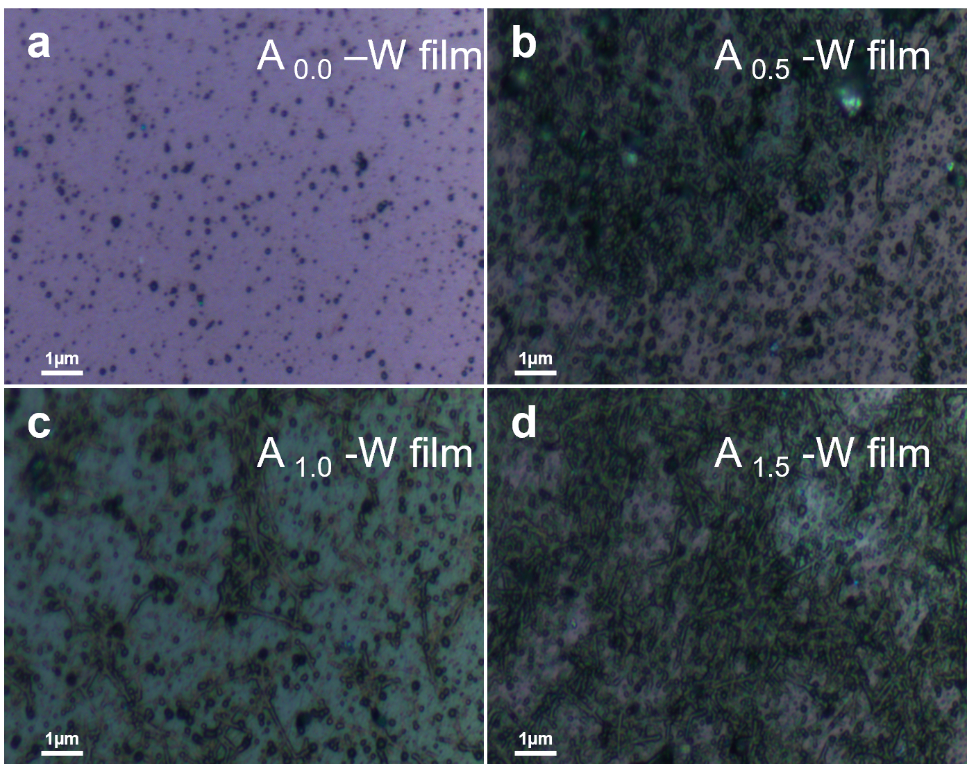


Fig. S14 Optical micrographs of A_x_-W films

**
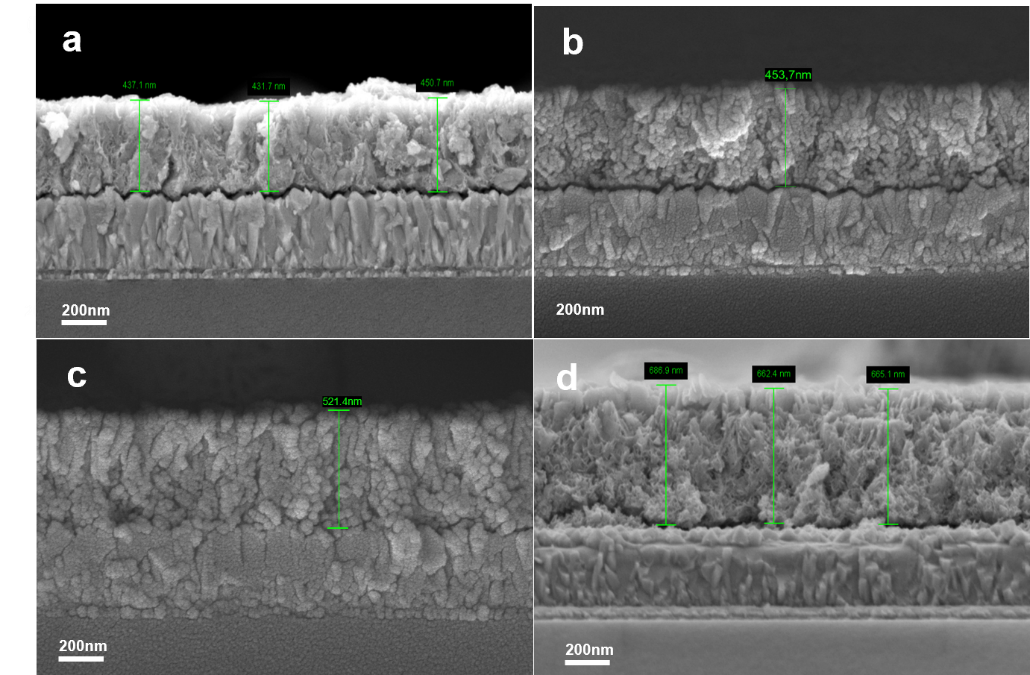
**

Fig. S15 SEM cross-section of A_x_-W films. (a) A_0.0_-W film growth 30 min section. (b) A_0.5_-W film growth 30 min section. (c) A_1.0_-W film growth 30 min section. (d) A_1.5_-W film growth 30 min section


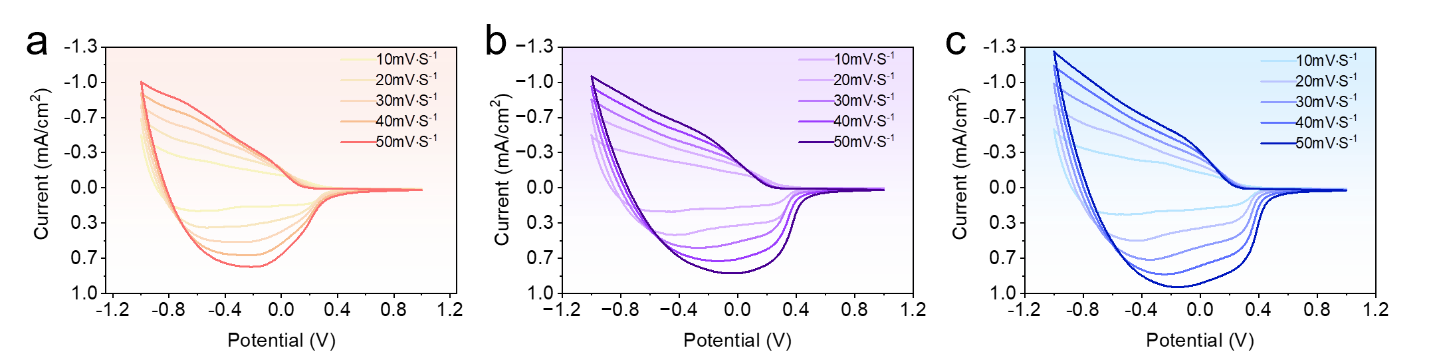


Fig. S16. CV plot of different scan rates of A_x_-W films. (a) A_0.0_-W film. (b) A_0.5_-W film. (c) A_1.5_-W film


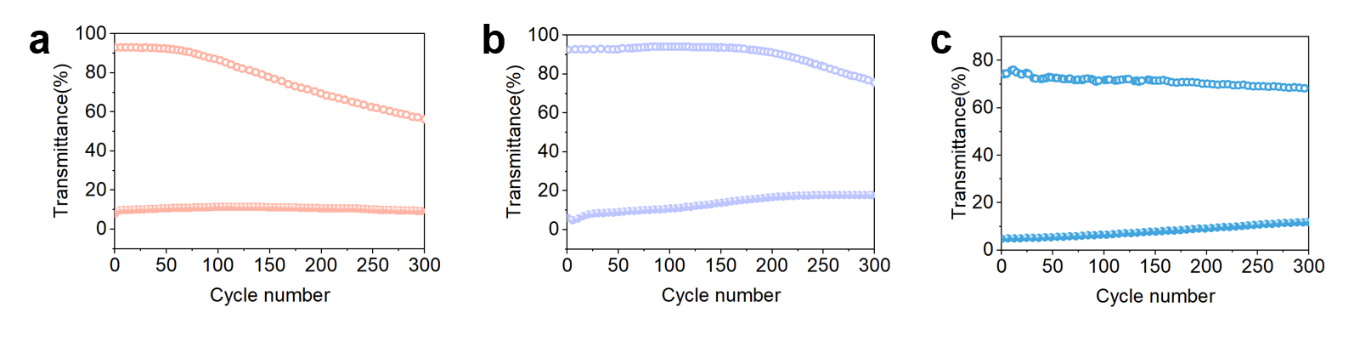


Fig. S17. Transmittance and number of cycles of A_x_-W films at 1100nm. (a) A_0.0_-W film. (b) A_0.5_-W film. (c) A_1.5_-W film

**
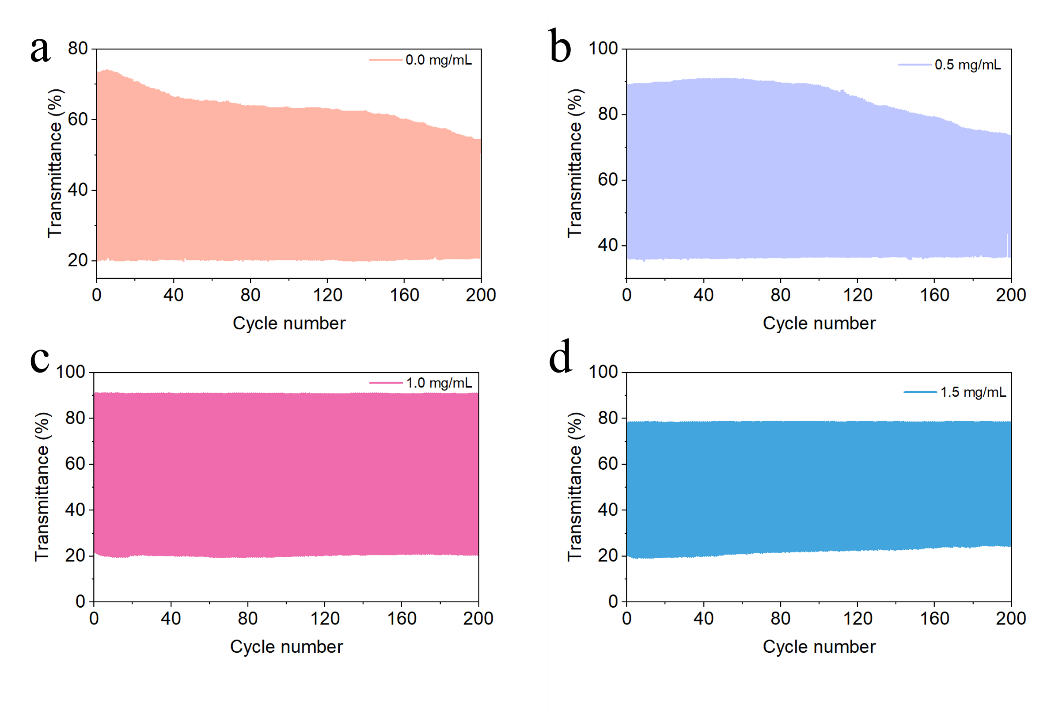
**

Fig. S18. Transmittance and number of cycles of A_x_-W films at 713nm. (a) A_0.0_-W film. (b) A_0.5_-W film. (c) A_1.0_-W film. (d) A_1.5_-W film

**
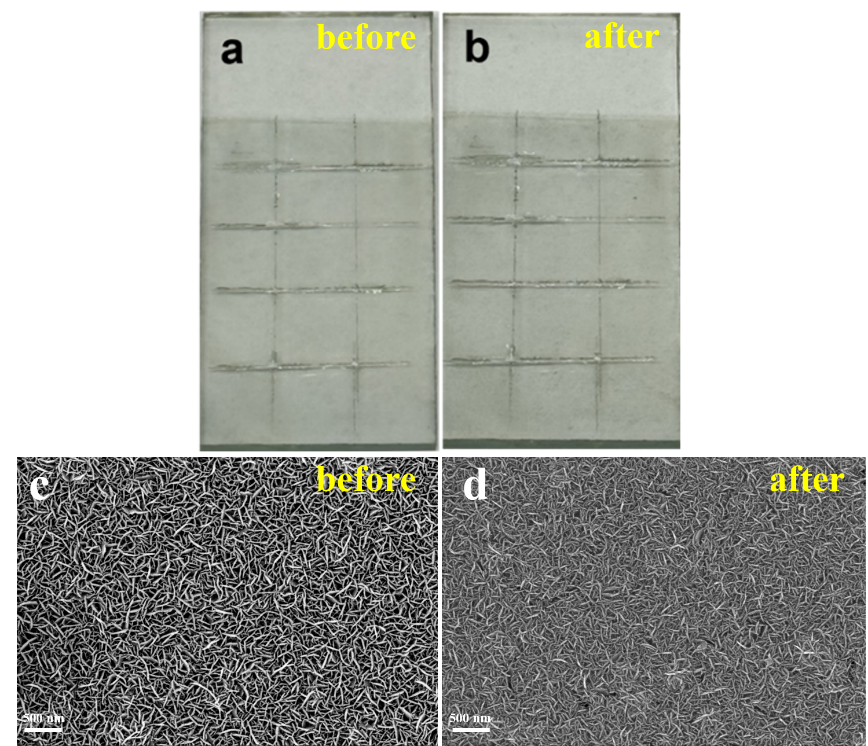
**

Fig. S19 a-b The adhesion test of the A_1.0_-W film. c-d The SEM morphologies of A_1.0_-W film before and after cycling

**
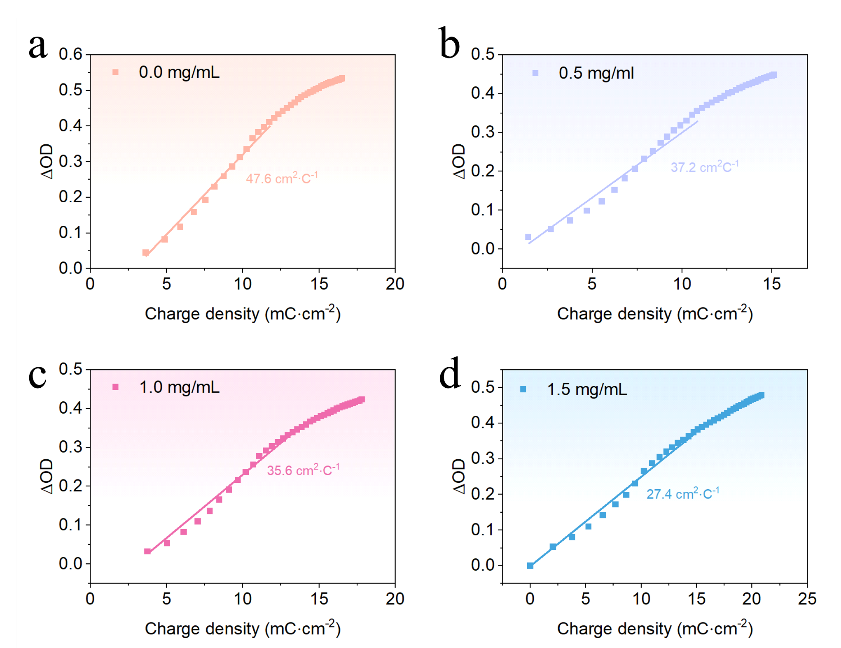
**

Fig. S20 Coloration efficiency plot of A_x_-W films at 713nm

**
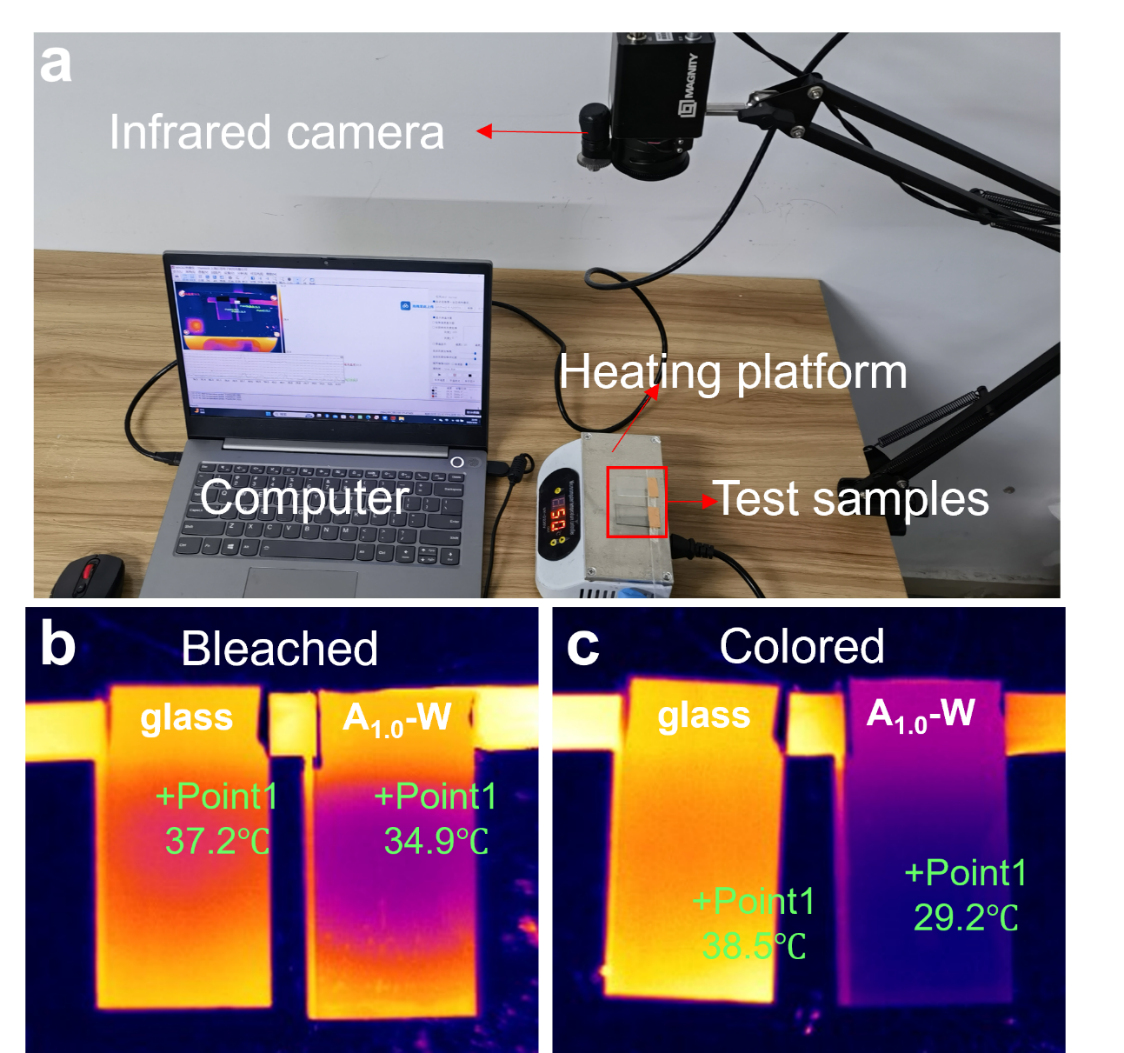
**

Fig. S21 Simulation of wall radiation test

**
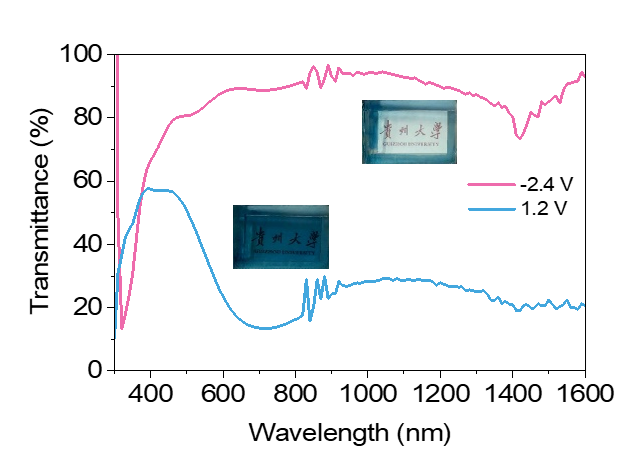
**

Fig. S22 Full transmittance spectrum of the device


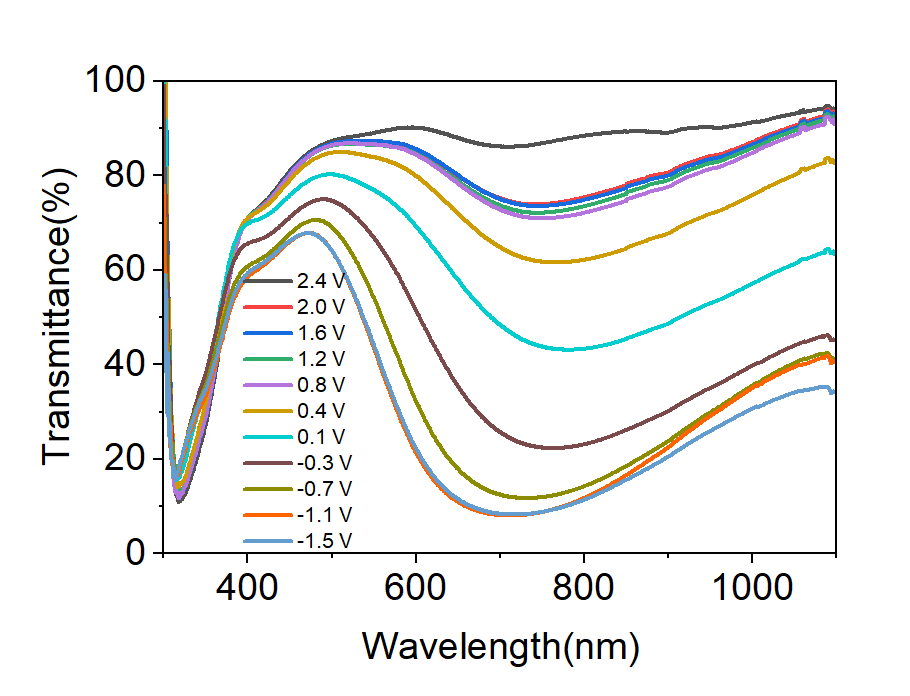


Fig. S23 The transmittance curves of the device at different voltages


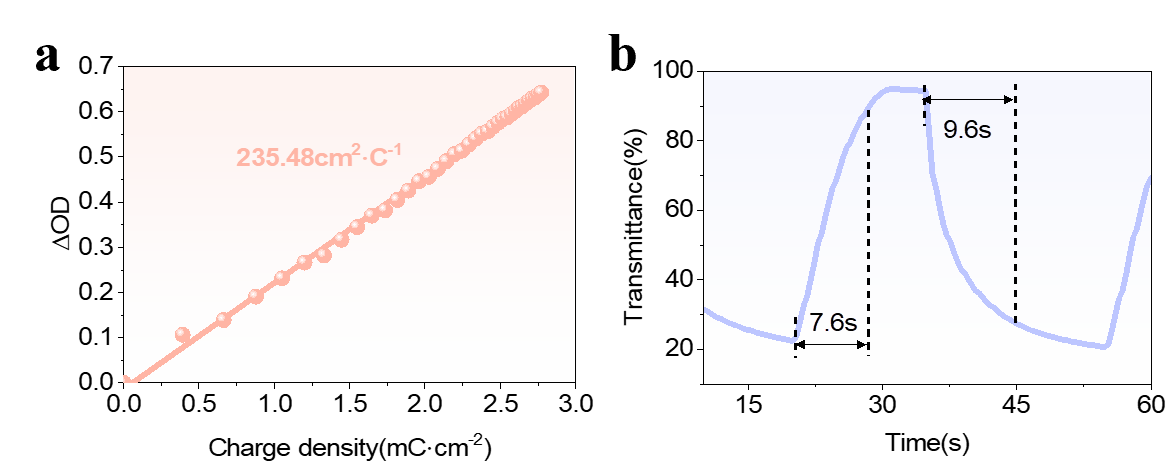


Fig. S24 The coloring efficiency and response time of the device at 1100 nm. a The coloring efficiency. b The response time


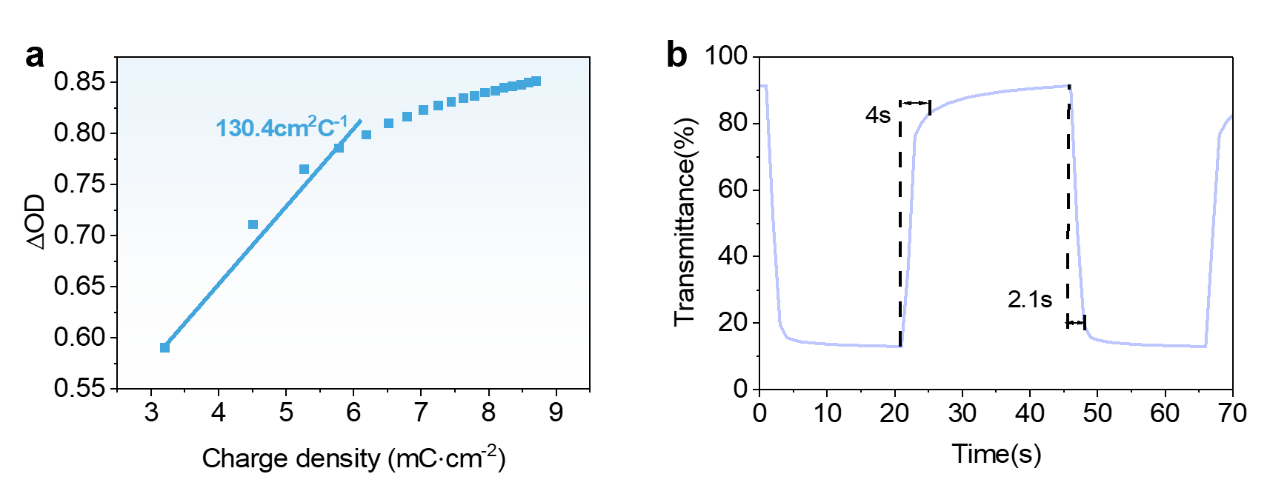


Fig. S25 The coloring efficiency and response time of the device at 713 nm. a The coloring efficiency. b The response time

**
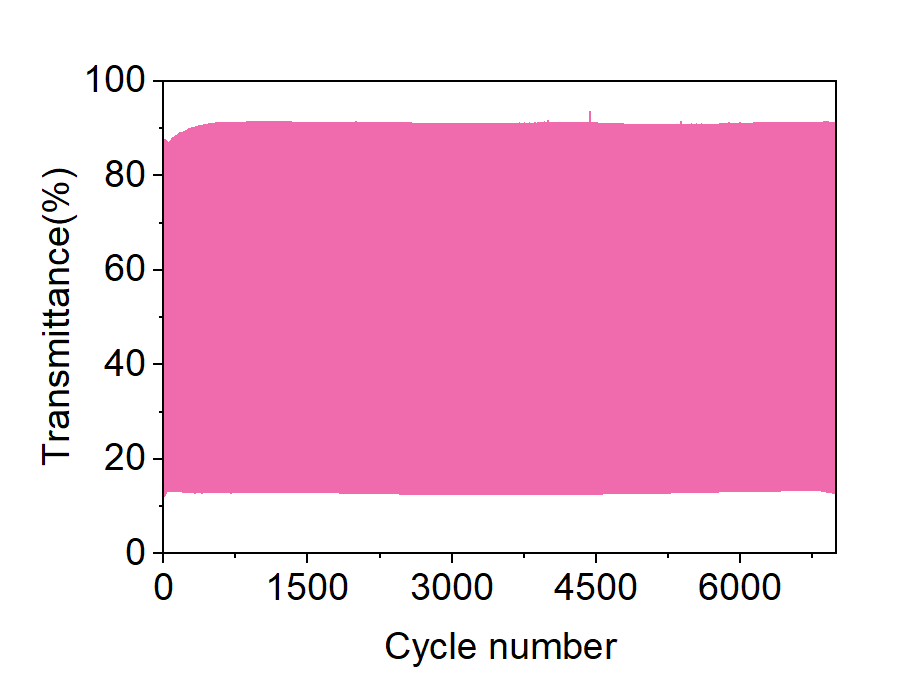
**

Fig. S26 The cycling data graph of device at 713 nm


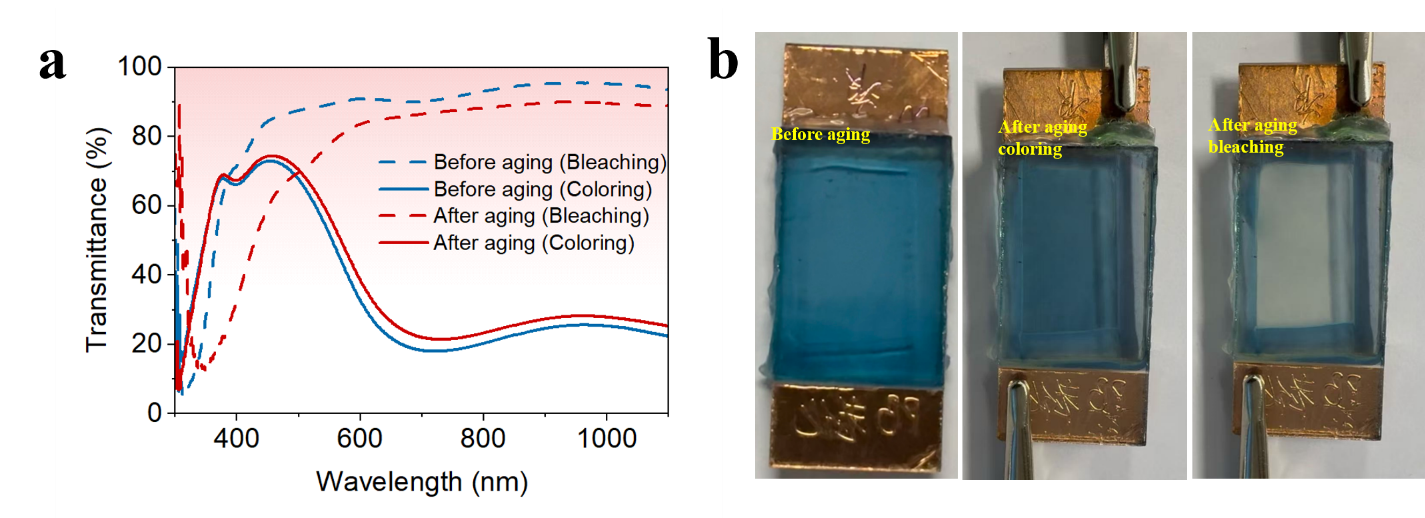


Fig. S27 a The full spectral transmittance before and after ECD aging. b Digital photos of the ECD before and after aging


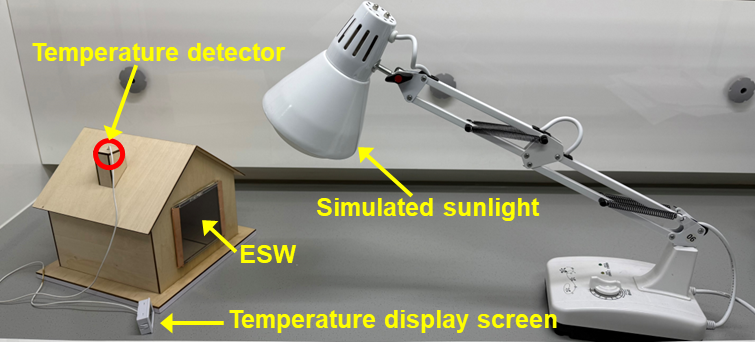


Fig. S28 The digital photographs of testing solar shielding capabilities of smart window


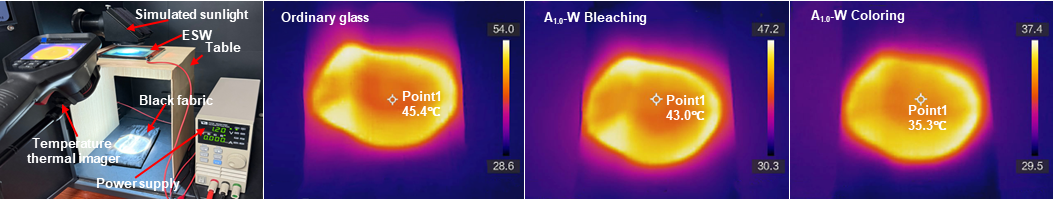


Fig. S29 Solar light simulation experiment and data graph

**
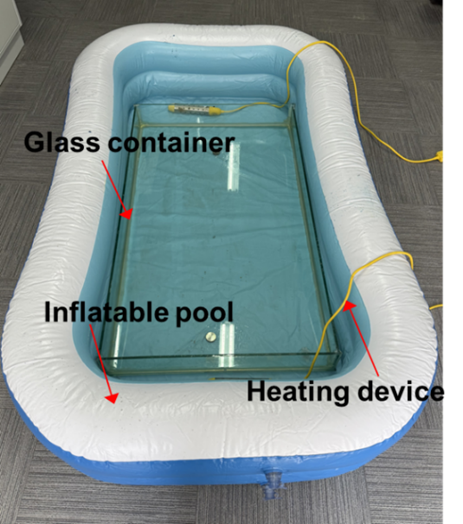
**

**Fig. S30** Composition of the facility for the preparation of large-scale films


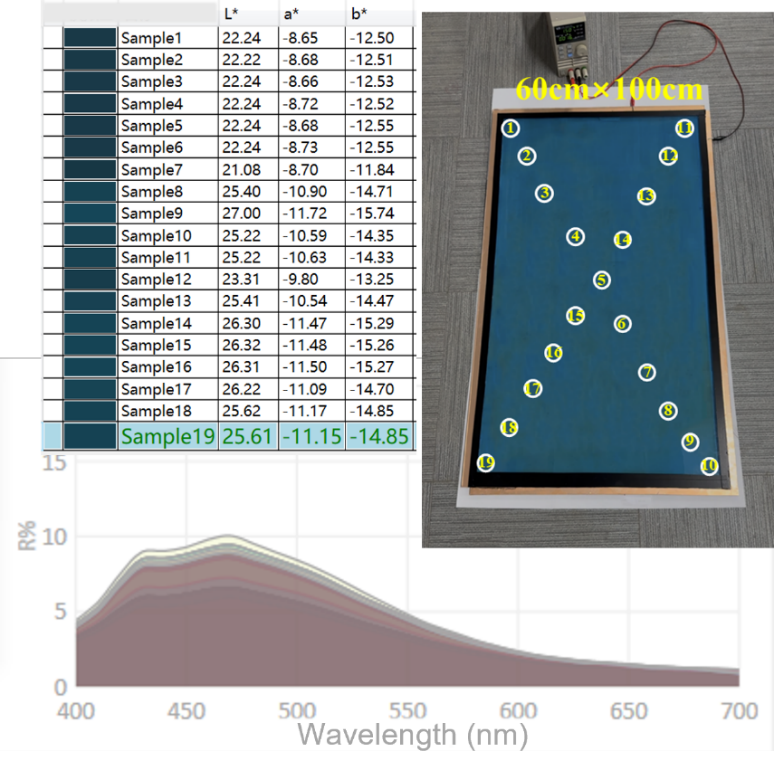


**Fig. S31** Three chromaticity coordinate parameters and spectral reflectance curves graphs of nineteen different point locations


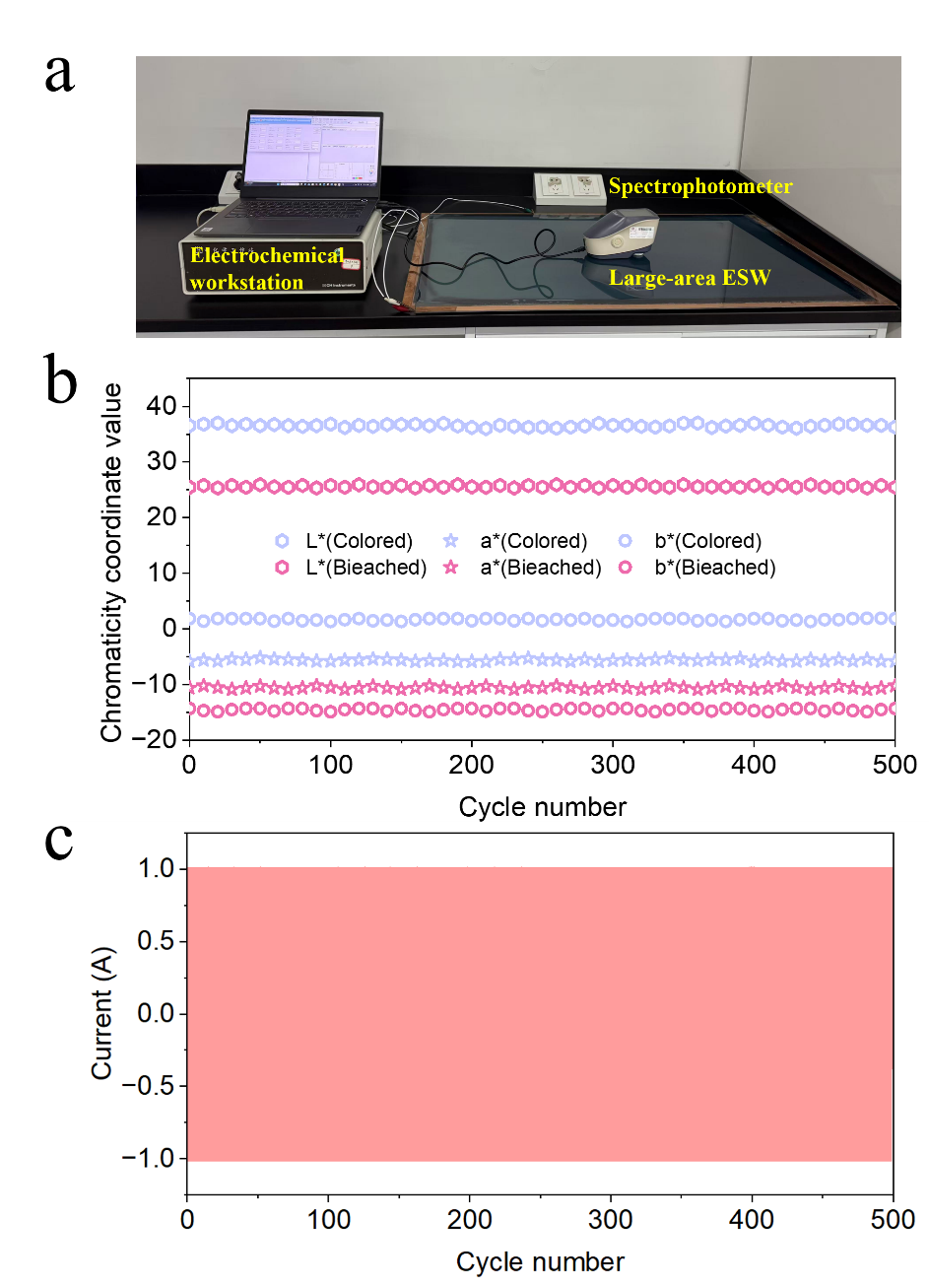


Fig. S32 a The Photograph of the facility used to test the cyclic stability of large-scale ESW. b The chromaticity coordinate value versus cycle number. c The current versus cycle number


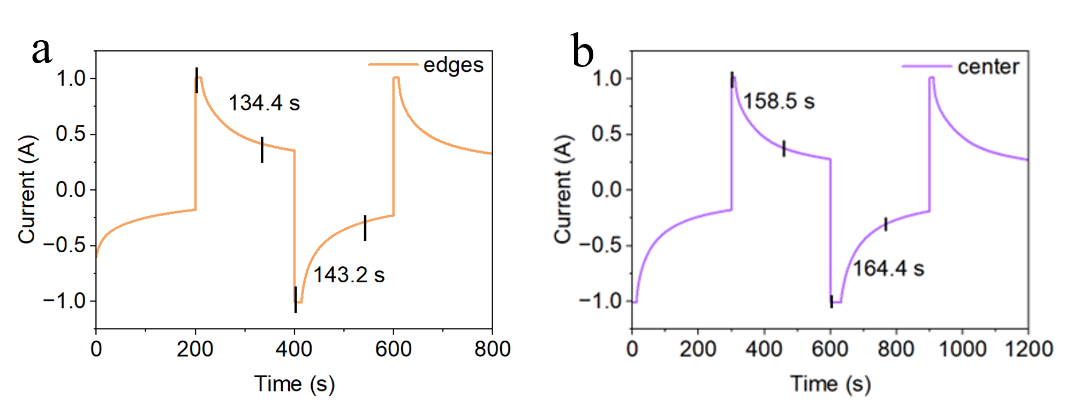


Fig. S33 The switching time of the large-scale ESW


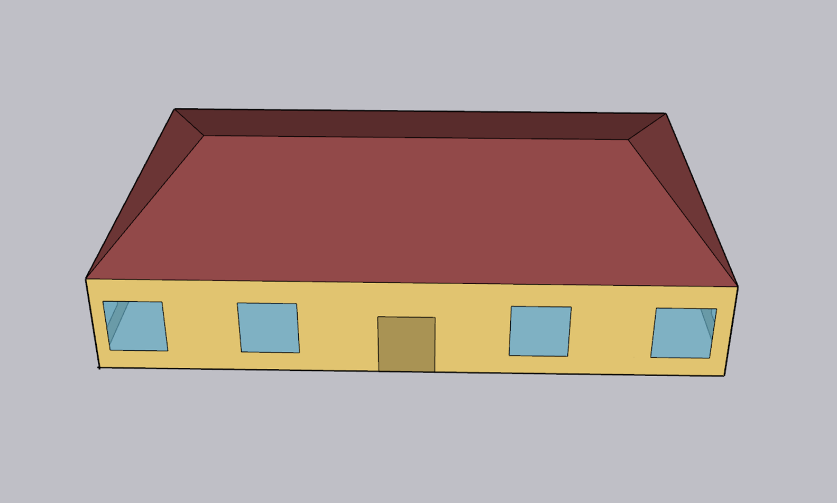


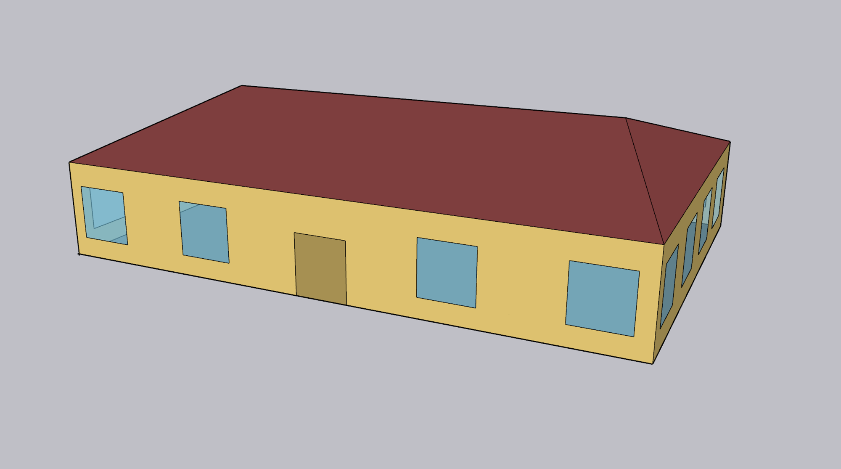


Fig. S34 The building model for energy simulation with the building size of 27 m×18 m×7 m, window size: 2.5 m×2.5 m


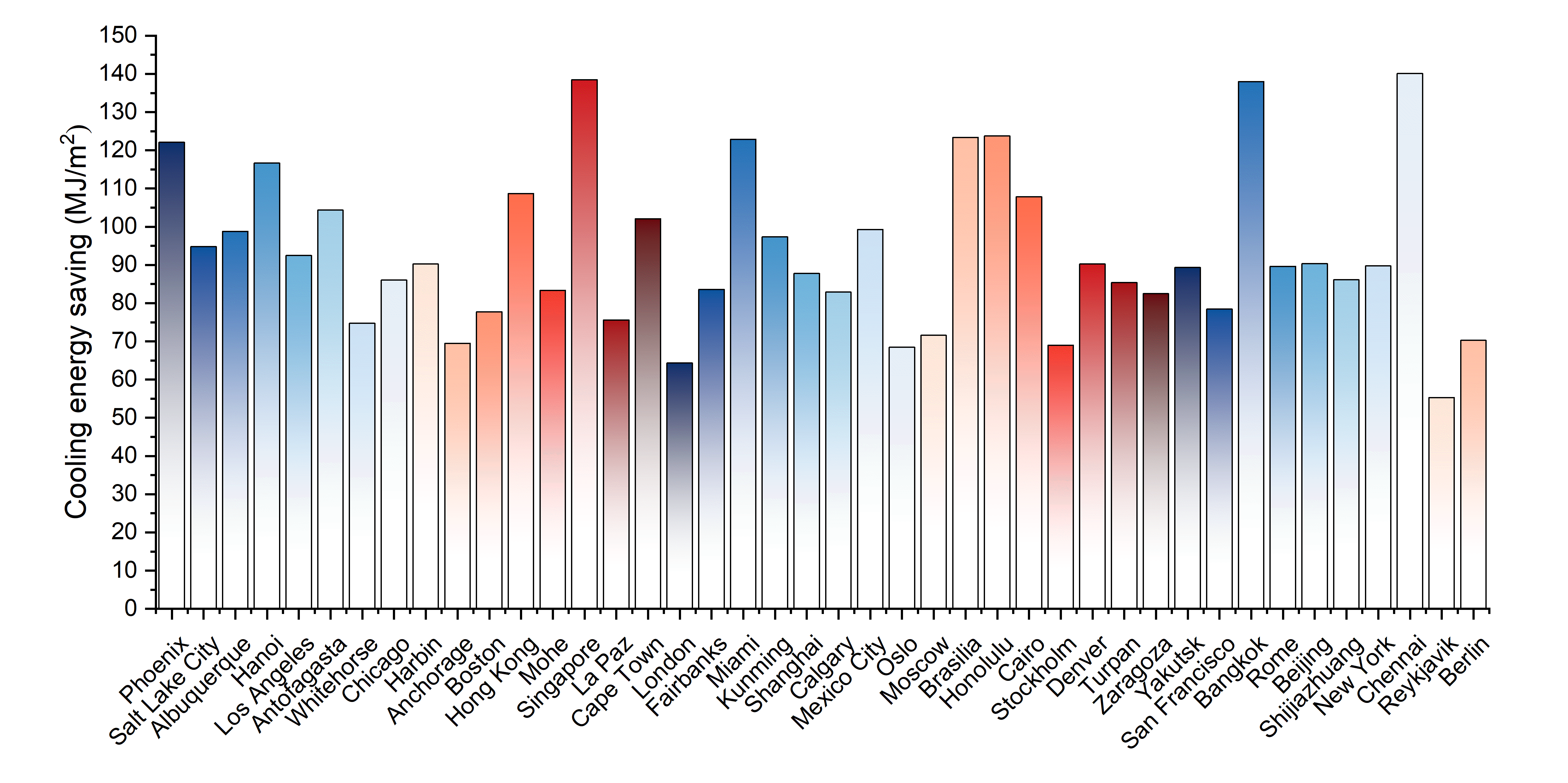


Fig. S35 The bar chart of energy conservation in different cities compared to traditional windows


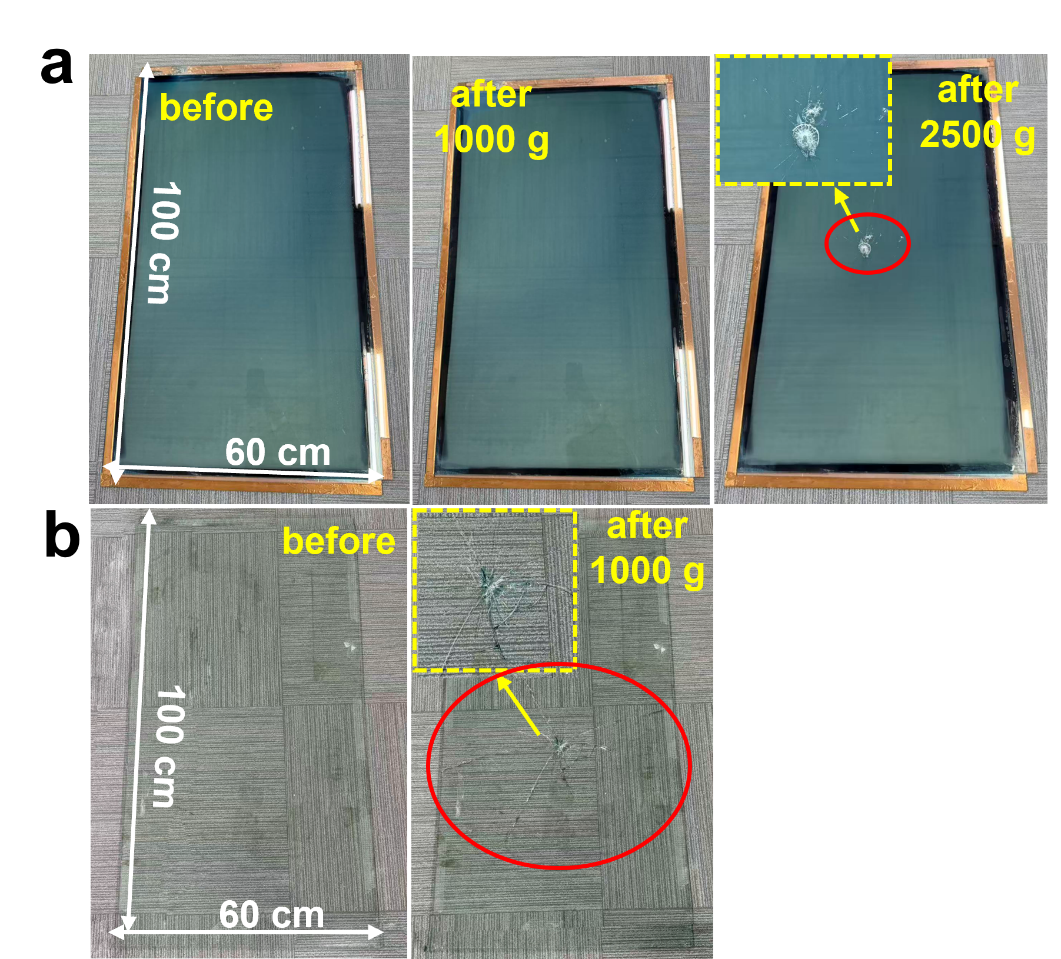


Fig. S36 a Digital image of ESW impact test. b Digital image of traditional glass impact test

Table S1 Research comparison of tungsten oxide-based electrochromic films

| **Materials** | **ΔT (%)** | **cycle times (cycles)** | **The total response time of the film (s)** |
| --- | --- | --- | --- |
| AP-WO_3_ SS7] | 75.5 | 1000 | 15.8 |
| WO_3-x_ [S8] | 64 | 450 | 8.4 |
| h-WO_3_ [S9] | 86 | 3000 | 10.6 |
| WO_3_ (NH_4_CF_3_SO_3_-TEP) [S10] | 76.1 | 1000 | **\** |
| WO_3_ (PVA-SN-H_3_PO_4_/LiTFSI) [S11] | 41 | 1000 | 60 |
| WO_3_·H_2_O (Mo, N) [S12] | 78 | 600 | 11 |
| Ti-doped WO_3_ [S13] | 90.3 | 1000 | 8.1 |
| Zn-WO_3_ [S14] | 50 | 240 | 41.5 |
| o-WO_3-x_ [S15] | 89.1 | 8000 | 24.7 |
| amorphous WO_3_ nanodots [S16] | 39 | **\** | 9.8 |
| P-WO_3_ [S17] | 65.4 | 3000 | 8.1 |
| WO_3-x_·nH_2_O(n < 2) [S18] | 74.2 | 20000 | 9.2 |
| a-WO_3-x_-OV [S19] | 80.3 | 8000 | 13.4 |
| WQD@MXene [S20] | 82.1 | 1000 | 7.7 |
| WO_3_-V-NiO [S21] | 88 | 21 | 75 |
| GDQDs/WO_3_ [S22] | 83.5 | 20000 | 21 |
| WO_3-y_ [S23] | 70 | 5000 | **\** |
| ITO-WO_3_ [S24] | 38 | 2000 | 14.1 |
| This work | 93.8 | 20,000 | 12.3 |

The symbol ‘\’ means no report on this aspect in the literatures.

Table S2 Research comparison of tungsten oxide-based electrochromic films

| **Material** | **ΔT of the**  **WO_3_ film** | **Film optical stability** | **ΔT of the ECD based WO_3_** | | **ECD optical stability** | **Maximum size of the ECD (**cm^2^) |
| --- | --- | --- | --- | --- | --- | --- |
| **Amorphous porous WO_3_** [S25] | 68.5%  at 633 nm | 75% retention after 1000 | 50%  at 633 nm | 60.2% retention after 1000 cycles  at 633 nm | | 12 |
| **Amorphous hydrated WO_3_·0.5H_2_O** [S7] | 66.1%  at 633 nm | 97% retention after 13000 cycles | 55%  at 633 nm | **\** | | 100 |
| **WO_3_/Al** [S21] | 88%  at 550 nm | 85% retention after 3000 cycles | 80%  at 550 nm | **\** | | 28 |
| **WO_3_/ITO/Al** [S26] | 84.89%  at 550 nm | **\** | 60%  at 550 nm | **\** | | 900 |
| **WH-rGO/ITO** [S27] | 43% at  700 nm | **\** | 24%  at 700 nm | **\** | | 25 |
| **a-WO_3_/FTO** [S28] | 70%  at 700 nm | 90% retention after 1000 cycles | 65%  at 700 nm | **\** | | 100 |
| **WO_3-x_·nH_2_O** [S18] | 74.2%  at 1100 nm | 94.7% retention after 20,000 cycles | 60.3%  at 708 nm  65.1%  at 1100 nm | 96.9% retention after 20000 cycles  at 1100 nm | | 6000 |
| **WO_3_·H_2_O** [S29] | **\** | **\** | 53.6%  at 650 nm | 82.5% retention after 2500 cycles  at 650 nm | | 110 |
| **MnO_2_ + WO_3_** [S30] | **\** | **\** | 67.3% | 85% retention after 3000 cycles | | 100 |
| **γ-WO_3_** [S31] | **\** | **\** | **\** | **\** | | 6.25 |
| **Gd-doped WO_3_** [S32] | 52.7%  at 633 nm | 81.9% retention after 2000 cycles | 50.3%  at 677 nm | 80.9% retention after 2000 cycles | | 1.5 |
| **WO_3_/xNC** [S33-S35] | 60.9%  at 633 nm | 91% retention after 2500 cycles | **\** | **\** | | **\** |
| **WO_3_\|AGF ECB** [S36] | **\** | **\** | 80.9%  at 700 nm | 94.4% retention after 8000 cycles | | 100 |
| **WO_3_/Nb_2_O_5_** [S37] | 78.4%  at 700 nm | 97.2% retention after 10000 cycles | 56.5%  at 700 nm | **\** | | 25 |
| **WO_3_·xH_2_O** [S33] | 74-88%  at 633 nm | 90% retention after 1500 cycles | 30-83%  at 633 nm | 80% retention after 500 cycles | | 25 |
| **WO_3_/Au/Zn^2+^/Zn** [S38] | **\** | **\** | **\** | 80.4% retention after 1000 cycles | | 48 |
| **WO_3_@Nd-Co_3_O_4_** [S39] | 79.3%  at 600 nm | 98.6% retention after 15000 s | 74.9%  at 600 nm | 92.8% retention after 4000 s | | 12 |
| **Ov-WO_3-x_/ZnO** [S40] | 74.5%  at 1000 nm | 40.4% retention after 2000 cycles | 60.4%  at 1000 nm | 40.4% retention after 2000 cycles | | 1600 |
| **This work** | 90.8%  at 1100 nm | 86.4% retention after 20,000 cycles | 75.1%  at 713 nm  78.9%  at 1100 nm | 80% retention after 8000 cycles  at 713 nm  79.4% retention after 10000 cycles  at 1100 nm | | 6000 |

The symbol ‘\’ means no report on this aspect in the literatures.

Table S3 Detailed information on the building model

| **Items** | **Specifications** |
| --- | --- |
| Window-to-wall ratio | 0.28 |
| Floor-to-ceiling height | 7.5 m |
| Glazing sill height | 0.9 m |
| Outer walls | Stucco/Concrete/Wall insulation/Gypsum |
| Interior walls | Gypsum board/Wall air space resistance/Gypsum board |
| Roof | Roof membrane/Roof insulation/Metal decking |
| Floor | Concrete/Carpet pad |

Table S4 Detailed information on the building model

| **Country** | **City** | **Climate Zone** |
| --- | --- | --- |
| Singapore | Singapore | Af |
| America | Honolulu | AM |
| America | Miami | Am |
| Vietnam | Ho Chi Minh City | Aw |
| Egypt | Cairo | Bwh |
| China | Shijiazhuang | Bsk |
| China | Turpan | Bwk |
| China | Shanghai | Cfa |
| Britain | London | Cfb |
| Iceland | Reykjavik | Cfc |
| Italy | Rome | Csa |
| Chile | Antofagasta | Csb |
| South Africa | Cape Town | Csc |
| China | Hong Kong | Cwa |
| China | Kunming | Cwb |
| Bolivia | La Paz | Cwc |
| America | Chicago | Dfa |
| Russia | Moscow | Dfb |
| America | Fairbanks | Dfc |
| Russia | Yakutsk | Dfd |
| America | Salt Lake City | Dsa |
| India | Chennai | Dsb |
| America | Anchorage | Dsc |
| China | Beijing | Dwa |
| Canada | Calgary | Dwb |
| China | Mohe | Dwc |
| America | Denver | BSK |
| Spain | Zaragoza | BSK |
| America | Phoenix | BSH |
| America | San Francisco | CSB |
| China | Harbin | DWB |
| Germany | Berlin | DFB |
| America | Boston | DFA |

Description of Supplementary Movies S1 and S2

**Movie S1** Real-time demonstration of the dynamic optical transition of electrochromic devices based on WO_3_ films

**Movie S2** Displays the smartphone user-friendly interface, enabling smooth switching between various ESW operating modes

**Supplementary References**

1. S.J. Clark, M.D. Segall, C.J. Pickard, P.J. Hasnip, M.I.J. Probert et al., First principles methods using CASTEP. Z. Für Kristallogr. Cryst. Mater. **220**(5–6), 567–570 (2005). <https://doi.org/10.1524/zkri.220.5.567.65075>
2. D. Vanderbilt, Soft self-consistent pseudopotentials in a generalized eigenvalue formalism. Phys. Rev. B **41**(11), 7892–7895 (1990). <https://doi.org/10.1103/physrevb.41.7892>
3. J.P. Perdew, K. Burke, M. Ernzerhof, Generalized gradient approximation made simple. Phys. Rev. Lett. **77**(18), 3865–3868 (1996). <https://doi.org/10.1103/physrevlett.77.3865>
4. S. Grimme, Semiempirical GGA-type density functional constructed with a long-range dispersion correction. J. Comput. Chem. **27**(15), 1787–1799 (2006). <https://doi.org/10.1002/jcc.20495>
5. H.J. Monkhorst, J.D. Pack, Special points for Brillouin-zone integrations. Phys. Rev. B **13**(12), 5188–5192 (1976). <https://doi.org/10.1103/physrevb.13.5188>
6. M. Tian, M. Lu, W. Wu, R. Zheng, Y. Tang et al., Towards a large-scale and high-performance smart window based on Prussian blue: a revolutionary two-dimensional-material assisted *in situ* growth preparation method utilizing MXene. J. Mater. Chem. A **12**(4), 2053–2069 (2024). <http://dx.doi.org/10.1039/d3ta06768h>
7. S. Zhang, Y. Peng, J. Zhao, Z. Fan, B. Ding et al., Amorphous and porous tungsten oxide films for fast-switching dual-band electrochromic smart windows. Adv. Opt. Mater. **11**(1), 2202115 (2023). <https://doi.org/10.1002/adom.202202115>
8. W. Wu, L. Wu, H. Ma, L. Wu, H. Wang et al., Electrochromic devices constructed with water-in-salt electrolyte enabling energy-saving and prolonged optical memory effect. Chem. Eng. J. **446**, 137122 (2022). <https://doi.org/10.1016/j.cej.2022.137122>
9. X. Tong, J. Wang, P. Zhang, P. Lei, Y. Gao et al., Insight into the structure–activity relationship in electrochromism of WO_3_ with rational internal cavities for broadband tunable smart windows. Chem. Eng. J. **470**, 144130 (2023). <https://doi.org/10.1016/j.cej.2023.144130>
10. J. Zhong, B. Huang, J. Song, X. Zhang, L. Du et al., Stable WO_3_ electrochromic system based on NH_4_^+^ hydrogen bond chemistry. Chem. Eng. J. **480**, 148098 (2024). <https://doi.org/10.1016/j.cej.2023.148098>
11. H. Xu, I. Haider, Y. Zheng, W. Li, S. Zhuiykov et al., Towards the Solid-State electrochromic devices: Platform based on transparent and flexible solid polymer electrolyte. Chem. Eng. J. **508**, 161116 (2025). <https://doi.org/10.1016/j.cej.2025.161116>
12. Z. Wang, X. Guo, F. Ma, H. Wang, C. Zhang et al., Heteroatom co-doping engineering endows tungsten oxide with highly efficient dual-band electrochromic performance. Chem. Eng. J. **509**, 161281 (2025). <https://doi.org/10.1016/j.cej.2025.161281>
13. Q. Meng, S. Cao, J. Guo, Q. Wang, K. Wang et al., Sol-gel-based porous Ti-doped tungsten oxide films for high-performance dual-band electrochromic smart windows. J. Energy Chem. **77**, 137–143 (2023). <https://doi.org/10.1016/j.jechem.2022.10.047>
14. R. Roy, R. Greeshma, A. Basith, R. Banerjee, A.K. Singh, Self-rechargeable aqueous Zn^2+/^K^+^ electrochromic energy storage device *via* scalable spray-coating integrated with Marangoni flow. Energy Storage Mater. **71**, 103680 (2024). <https://doi.org/10.1016/j.ensm.2024.103680>
15. M. Chen, J. Deng, H. Zhang, X. Zhang, D. Yan et al., Advanced dual-band smart windows: inorganic all-solid-state electrochromic devices for selective visible and near-infrared modulation. Adv. Funct. Mater. **35**(3), 2413659 (2025). <https://doi.org/10.1002/adfm.202413659>
16. P. Liu, B. Wang, C. Wang, L. Ma, W. Zhang et al., Amorphous tungsten oxide nanodots for chromatic applications. Adv. Funct. Mater. **34**(34), 2400760 (2024). <https://doi.org/10.1002/adfm.202400760>
17. W.C. Poh, A.L. Eh, W. Wu, X. Guo, P.S. Lee, Rapidly photocurable solid-state poly(ionic liquid) ionogels for thermally robust and flexible electrochromic devices. Adv. Mater. **34**(51), e2206952 (2022). <https://doi.org/10.1002/adma.202206952>
18. M. Tian, R. Zheng, C. Jia, Bridging to commercialization: record-breaking of ultra-large and superior cyclic stability tungsten oxide electrochromic smart window. Adv. Mater. **37**(3), 2409790 (2025). <https://doi.org/10.1002/adma.202409790>
19. M. Chen, X. Zhang, D. Yan, J. Deng, W. Sun et al., Oxygen vacancy modulated amorphous tungsten oxide films for fast-switching and ultra-stable dual-band electrochromic energy storage smart windows. Mater. Horiz. **10**(6), 2191–2203 (2023). <http://dx.doi.org/10.1039/d2mh01472f>
20. Q. Zhao, J. Wang, J. Sun, C. Bao, X. Chen et al., A biomimetic alveoli-in-lung-structured electrode: robustly anchored tungsten oxide quantum dot on Ti3C2 MXene for multifunctional sodium-ion-based electrochromic devices. ENERGY ENVIRONMENTAL Mater. **8**(1), e12790 (2025). <https://doi.org/10.1002/eem2.12790>
21. M.K. Ganesha, H. Hakkeem, A.K. Singh, Redox potential based self-powered electrochromic devices for smart windows. Small **20**(42), 2403156 (2024). <https://doi.org/10.1002/smll.202403156>
22. W. Wu, M. Tian, Y. Tang, C. Song, R. Zheng et al., Revolutionizing dual-band modulation and superior cycling stability in GDQDs-doped WO_3_ electrochromic films for advanced smart window applications. Small **21**(6), 2407708 (2025). <https://doi.org/10.1002/smll.202407708>
23. Z. Zhang, H. Mo, R. Li, X. Zhou, Z. Lin et al., The counterbalancing role of oxygen vacancy between the electrochromic properties and the trapping effect passivation for amorphous tungsten oxide films. Small Sci. **4**(3), 2300219 (2024). <https://doi.org/10.1002/smsc.202300219>
24. H. Najafi-Ashtiani, M.M. Bilek, B. Akhavan, Tungsten oxide thin films for electrochromic applications: pulse width-controlled deposition by high-power impulse magnetron sputtering. Adv. Eng. Mater. **26**(6), 2301378 (2024). <https://doi.org/10.1002/adem.202301378>
25. D. Zhuang, Z. Zhang, J. Weng, J. Wang, H. Zhang et al., Amorphous hydrated tungsten oxides with enhanced pseudocapacitive contribution for aqueous zinc-ion electrochromic energy storage. Adv. Energy Mater. **14**(40), 2402603 (2024). <https://doi.org/10.1002/aenm.202402603>
26. M.K. Ganesha, I. Mondal, A.K. Singh, G.U. Kulkarni, Fabrication of large-area, affordable dual-function electrochromic smart windows by using a hybrid electrode coated with an oxygen-deficient tungsten oxide ultrathin porous film. ACS Appl. Mater. Interfaces **15**(15), 19111–19120 (2023). <https://doi.org/10.1021/acsami.2c22638>
27. P. Dutta, M. Verma, M.S. Paliwal, I. Mondal, M.K. Ganesha et al., Dual-functional electrochromic smart window using WO_3_·H_2_O-rGO nanocomposite ink spray-coated on a low-cost hybrid electrode. ACS Appl. Mater. Interfaces **15**(49), 57304–57313 (2023). <http://dx.doi.org/10.1021/acsami.3c11337>
28. W. Cheng, J. He, K.E. Dettelbach, N.J.J. Johnson, R.S. Sherbo et al., Photodeposited amorphous oxide films for electrochromic windows. Chem **4**(4), 821–832 (2018). <https://doi.org/10.1016/j.chempr.2017.12.030>
29. Z. Bi, X. Li, Y. Chen, X. He, X. Xu et al., Large-scale multifunctional electrochromic-energy storage device based on tungsten trioxide monohydrate nanosheets and Prussian white. ACS Appl. Mater. Interfaces **9**(35), 29872–29880 (2017). <http://dx.doi.org/10.1021/acsami.7b08656>
30. C. Zhang, S. Li, R. Wu, S. Wu, X. Wang et al., Robust MnO(2)-WO(3) complementary electrochromic device enabled by reversible electrodeposition of MnO(2). Nano Lett. **24**(51), 16360–16367 (2024). <https://doi.org/10.1021/acs.nanolett.4c04849>
31. T. Rocca, A. Gurel, D. Schaming, B. Limoges, V. Balland, Multivalent-ion versus proton insertion into nanostructured electrochromic WO_3_ from mild aqueous electrolytes. ACS Appl. Mater. Interfaces **16**(18), 23567–23575 (2024). <http://dx.doi.org/10.1021/acsami.4c02152>
32. Y. Yin, Y. Zhu, P. Liao, X. Yuan, J. Jia et al., Co-sputtering construction of Gd-doped WO_3_ nano-stalagmites film for bi-funcional electrochromic and energy storage applications. Chem. Eng. J. **487**, 150615 (2024). <https://doi.org/10.1016/j.cej.2024.150615>
33. M. Ma, S. Jia, C. Bian, W. Yang, Y. Zhong et al., Wide-temperature adaptive electrochromic device enabled by deep eutectic solvent-based electrolytes. Adv. Funct. Mater. e20971 (2025). <https://doi.org/10.1002/adfm.202520971>
34. W.-T. Xie, M.-Q. Xiao, L. Huang, Q.-Q. Qiu, H. Li et al., Ant nest-like WO_3_ films for improving electrochromic and energy-storage dual-functional performance by the surface modification of N-doped carbon. Tungsten **7**(2), 393–404 (2025). <https://doi.org/10.1007/s42864-024-00305-w>
35. S. Jia, X. Guo, Y. Lv, H. Zhou, J. Wang et al., Ultrathin WO_3_·H_2_O nanosheets derived from the *Aurivillius* phase for high-performance dual-band electrochromic smart windows. Mater. Horiz. **12**(23), 10147–10156 (2025). <http://dx.doi.org/10.1039/d5mh01354b>
36. Y. Chen, J. Wan, C. Zhang, X. Tang, L. Chen et al., Enhanced high-energy-density WO_3_-based electrochromic batteries *via* acid-modified graphite foil cathode. Nano Res. **18**(12), 94907799 (2025). <https://doi.org/10.26599/nr.2025.94907799>
37. Y. Chen, W. Jiang, C. Zhang, Q. Shen, D. Li et al., Enhanced electrochromic performance and cycling stability *via* built-In electric field in WO_3_/Nb_2_O_5_ heterostructure. Adv. Funct. Mater. e19286 (2025). <https://doi.org/10.1002/adfm.202519286>
38. D. Yan, L. Liu, T. Guo, Z. Zhang, M. Wang et al., High-performance reflective-type Zn-ions electrochromic devices toward visible-to-infrared broadband dynamic regulation. Chem. Eng. J. **504**, 159117 (2025). <https://doi.org/10.1016/j.cej.2024.159117>
39. P.J. Morankar, R.U. Amate, A.M. Teli, M.K. Bhosale, S.A. Beknalkar et al., WO_3_@Nd-Co_3_O_4_ bilayer composites for functionally coupled electrochromic energy storage materials. Chem. Eng. J. **524**, 169630 (2025). <https://doi.org/10.1016/j.cej.2025.169630>
40. M. Chen, X. Zhang, W. Sun, Y. Xiao, H. Zhang et al., A dual-responsive smart window based on inorganic all-solid-state electro- and photochromic device. Nano Energy **123**, 109352 (2024). <https://doi.org/10.1016/j.nanoen.2024.109352>
